# FABP4 as a key determinant of metastatic potential of ovarian cancer

**DOI:** 10.1038/s41467-018-04987-y

**Published:** 2018-07-26

**Authors:** Kshipra M. Gharpure, Sunila Pradeep, Marta Sans, Rajesha Rupaimoole, Cristina Ivan, Sherry Y. Wu, Emine Bayraktar, Archana S. Nagaraja, Lingegowda S. Mangala, Xinna Zhang, Monika Haemmerle, Wei Hu, Cristian Rodriguez-Aguayo, Michael McGuire, Celia Sze Ling Mak, Xiuhui Chen, Michelle A. Tran, Alejandro Villar-Prados, Guillermo Armaiz Pena, Ragini Kondetimmanahalli, Ryan Nini, Pranavi Koppula, Prahlad Ram, Jinsong Liu, Gabriel Lopez-Berestein, Keith Baggerly, Livia S. Eberlin, Anil K. Sood

**Affiliations:** 1 Department of Gynecologic Oncology and Reproductive Medicine, The University of Texas MD Anderson Cancer Center, Houston, Texas 77030 USA; 2 Department of Obstetrics and Gynecology, Medical College of Wisconsin, Milwaukee, WI 53226 USA; 3 Department of Chemistry, The University of Texas at Austin, Austin, TX 78712 USA; 4 Department of Pathology and Institute of RNA Medicine, Beth Israel Deaconess Medical Center Cancer Center, Harvard Medical School, Boston, MA 02215 USA; 5 Center for RNA Interference and Non-Coding RNA, The University of Texas MD Anderson Cancer Center, Houston, 77030, Texas USA; 6 Department of Experimental Therapeutics, The University of Texas MD Anderson Cancer Center, Houston, TX 77030 USA; 7 Martin-Luther-University Halle-Wittenberg, Institute of Pathology, 06112 Halle (Saale), Germany; 8 Department of Pharmacology, Ponce Health Sciences University, Ponce, 00716 Puerto Rico; 9 The University of Texas at Austin, Austin, TX 78712 USA; 10 Graduate School of Biomedical Sciences, The University of Texas MD Anderson Cancer Center, Houston, TX 77030 USA; 11 Department of Experimental Radiation Oncology, The University of Texas MD Anderson Cancer Center, Houston, TX 77030 USA; 12 Department of Systems Biology, The University of Texas MD Anderson Cancer Center, Houston, TX 77030 USA; 13 Department of Pathology, Division of Pathology and Laboratory Medicine, The University of Texas MD Anderson Cancer Center, Houston, TX 77030 USA; 14 Department of Bioinformatics and Computational Biology, The University of Texas MD Anderson Cancer Center, Houston, TX 77030 USA; 15 Department of Cancer Biology, The University of Texas MD Anderson Cancer Center, Houston, TX 77030 USA

## Abstract

The standard treatment for high-grade serous ovarian cancer is primary debulking surgery followed by chemotherapy. The extent of metastasis and invasive potential of lesions can influence the outcome of these primary surgeries. Here, we explored the underlying mechanisms that could increase metastatic potential in ovarian cancer. We discovered that FABP4 (fatty acid binding protein) can substantially increase the metastatic potential of cancer cells. We also found that miR-409-3p regulates FABP4 in ovarian cancer cells and that hypoxia decreases miR-409-3p levels. Treatment with DOPC nanoliposomes containing either miR-409-3p mimic or *FABP4* siRNA inhibited tumor progression in mouse models. With RPPA and metabolite arrays, we found that FABP4 regulates pathways associated with metastasis and affects metabolic pathways in ovarian cancer cells. Collectively, these findings demonstrate that FABP4 is functionally responsible for aggressive patterns of disease that likely contribute to poor prognosis in ovarian cancer.

## Abstract (web summary)

In ovarian cancer, metastatic phenotype may impact surgical outcomes. Here, the authors show miR-409-3p regulates FABP4 which can increase metastatic potential of ovarian cancer, and treatment with DOPC nanoliposomes containing either miR-409--3p mimic or FABP4 siRNA inhibits tumor progression in mice.

## Introduction

Primary cytoreductive surgery followed by adjuvant chemotherapy is the standard treatment for ovarian cancer. Several reports have established a link between residual disease after surgery and shorter overall and progression-free survival, as well as poor response to adjuvant chemotherapy^1–3^. Incomplete resection can result from the presence of numerous, dense nodules that simply cannot be removed, distant tumor metastasis, location of tumor near critical organs (e.g., porta hepatis), and extensive mesenteric involvement. Thus, while a surgeon’s skill is important, residual disease could occur because the intrinsic biology of the tumor creates a highly metastatic and infiltrative disease pattern, making complete resection infeasible^3,4^. A large retrospective study showed that initial disease distribution could also affect patient survival despite aggressive cytoreductive efforts^5^. Considering the impact of residual disease on patient survival, many studies are now focusing on the development of predictive models for residual disease. However, currently there is little understanding of the biological mechanisms that could lead to residual disease in ovarian cancer. To address this knowledge gap, in the current study, we explored the underlying tumor biology responsible for the aggressive pattern of tumor metastasis.

We have previously shown that high expression of fatty acid binding protein 4 (FABP4) can be a reliable molecular predictor of residual disease in high-grade serous ovarian cancer^4^. Hence, we decided to investigate the mechanisms that lead to upregulation of FABP4 in ovarian cancer, as well as its downstream effects that lead to metastasis. FABP4 is implicated in atherosclerosis, diabetes, inflammatory response, and angiogenesis^6–12^. In prostate and ovarian cancers, FABP4 acts as a key mediator between adipocytes and cancer progression^13,14^. However, FABP4 regulation and the functional implications of FABP4 overexpression in increasing the metastatic potential of ovarian cancer cells remain to be investigated.

Here, we examined the biological effects of increased FABP4 expression in ovarian cancer. Through extensive bioinformatics analyses, we identified hypoxia mediated downregulation of miR-409-3p as a key regulator of FABP4. *FABP4* siRNA or a miR-409-3p mimic led to significant inhibition of tumor metastasis. Downstream of FABP4, we observed that FABP4 can affect several metabolites as well as metastasis-related pathways in ovarian cancer. Collectively, this study provides an understanding of the causal biology of metastatic potential in ovarian cancer.

## Results

### Effects of FABP4 on tumor progression

**Fig. 1:**
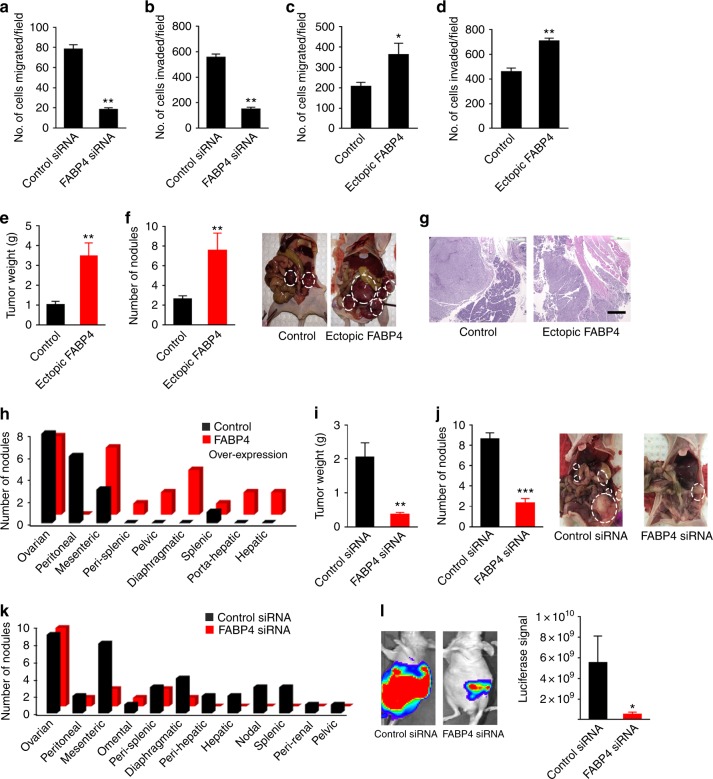
Effects of FABP4 on ovarian tumor progression. **a**, **b** Effect of knockdown of FABP4 on the **a** migration and **b** invasion of ovarian cancer cells (HeyA8 MDR). **p < 0.01. Effect of increased expression of FABP4 on the **c** migration and **d** invasion of cancer cells (A2780-ip1). *p < 0.05. **p < 0.01. **e** Aggregate mass of tumors in orthotopic mouse models involving A2780-ip1 cells transfected with control and FABP4 ectopic expression vectors (*n* = 10 mice per group, Student *t* test). **p < 0.01. **f** Effect of ectopically expressed FABP4 on the number of nodules in orthotopic mouse models involving A2780-control and A2780-FABP4 ectopic expression (*n* = 10 mice per group, Student *t* test). **p < 0.01. Representative images of nodules are shown on the right. **g** Representative hematoxylin and eosin-stained sections of tumor tissues from mice injected with control A2780-ip1 cells or A2780-ip1 cells transfected with FABP4-expressing vector. **h** Distribution of metastatic nodules present in the groups. **i** Aggregate mass of tumors in orthotopic mouse models involving HeyA8 MDR cells. Mice were treated with control or *FABP4* siRNA incorporated in DOPC liposomes (*n* = 10 mice per group). **p < 0.01. **j** Effect of knockdown of FABP4 on the number of metastatic nodules in HeyA8 MDR orthotopic mouse models. ***p < 0.001. Representative images are shown on the right. **k** Distribution of metastatic nodules present in individual mice after treatment with control or *FABP4* siRNA incorporated in DOPC liposomes. **l** Representative images of metastatic lesions revealed by luciferase imaging, and quantitative assessment of luciferase signal from mice treated with control or *FABP4* siRNA encapsulated in DOPC liposomes. *p < 0.05. Bars and errors bars represent the means and the corresponding SEMs for *n* > 3

To investigate the functional role of FABP4 in ovarian cancer progression, we first knocked down *FABP4* in HeyA8 MDR ovarian cancer cells (which have high FABP4 expression) using small interfering RNA (siRNA). FABP4 silencing led to a significant reduction in both migration and invasion of HeyA8 MDR cancer cells (Fig. 1a, b, Supplementary Fig. 1a and b) (*p* < 0.01). Conversely, ectopic expression of FABP4 in A2780-ip1 ovarian cancer cells led to increased migration (*p* < 0.05; Fig. 1c) and invasion of cancer cells (*p* < 0.01; Fig. 1d, Supplementary Fig. 1c and d). The effect was consistent when tested using a second siRNA sequence against FABP4 (Supplementary Fig. 1e and f), as well as another ovarian cancer cell line (Ovcar 5; Supplementary Fig. 1g and h).

Next, we examined the effect of FABP4 on tumor progression in orthotopic mouse models of ovarian cancer. We first transfected A2780-ip1 cells that have low endogenous FABP4 levels with a lentivirus expressing either a control vector or FABP4-expressing vector. We then injected the cells into the ovaries of athymic nude mice. This orthotopic model mimics the ovarian cancer metastasis pattern seen in patients^15,16^. Compared with the control mice, mice injected with cells ectopically expressing FABP4 had a significantly higher tumor burden (~3-fold, *p* < 0.01; Fig. 1e), as well as an increased number of distant metastatic nodules (~3-fold, *p* < 0.01; Fig. 1f). We examined hematoxylin and eosin-stained sections of tumor nodules from both groups. In the control group, the cancer cells did not invade normal organs (muscle, pancreas), however, after ectopic expression of FABP4, the cancer cells were found to be deeply invading the normal tissues (Fig. 1g and Supplementary Fig 1i). The tumor metastasis rate with FABP4 overexpression was significantly higher at sites such as the diaphragm, liver, and pelvis, whereas tumors in the control mice were mainly observed at the site of injection (ovary) and in the mesentery (Fig. 1h). Expression of FABP4 was examined in the tumor sections using immunohistochemistry, and expression of FABP4 was higher in sections from the ectopic FABP4 group than in those from the control group (Supplementary Fig. 1j). We also noted a positive correlation between the expression of FABP4 and the invasive capacity of ovarian cancer cell lines in vitro (Supplementary Fig 1k). Cell lines with higher FABP4 expression (HeyA8 MDR and Ovcar 5) had higher invasive potential compared to A2780 and SKOV3 cells.

We next investigated the effects of FABP4 knockdown in orthotopic mouse models of ovarian cancer. We first injected HeyA8 MDR-Luc cells into the ovaries of nude mice. Seven days after cell injection, DOPC nanoliposomes containing control siRNA or *FABP4* siRNA were administered intraperitoneally twice per week. The mice that received *FABP4* siRNA had a significant decrease in aggregate tumor weight (80%, *p* < 0.01) as well as number of metastatic nodules (75%, *p* < 0.01) compared with control mice (Fig. 1i, j). The effect was also observed in the pattern of metastasis. The treatment group had a significantly lower rate of metastasis, and the control group had widespread metastases to the diaphragm, liver, spleen, lymph node, and pelvic areas (Fig. 1k). Bioluminescence images also supported the finding that *FABP4* siRNA led to decreased metastasis in treated mice (Fig. 1l). The treatment did not affect mouse body weight (Supplementary Fig. 2a). We then checked expression of FABP4 in the tumors collected from the control and treatment groups. FABP4 expression was significantly decreased after treatment with *FABP4* siRNA, as observed in Western blot analysis (Supplementary Fig. 2b) and immunohistochemical staining (Supplementary Fig. 2c).

We also examined an additional orthotopic mouse model in which Ovcar 5 cells were injected directly into the ovary. Similar to the previous experiment, *FABP4* siRNA treatment resulted in significant reduction in tumor weight (*p* < 0.05; Supplementary Fig. 2d) and the number of nodules (*p* < 0.05; Supplementary Fig. 2e). Metastasis was also reduced at various sites, including the peritoneum, mesentery, and diaphragm (Supplementary Fig. 2f). The treatment had no effect on the body weight of the mice (Supplementary Fig. 2g).

### Upstream regulation of FABP4

**Fig. 2:**
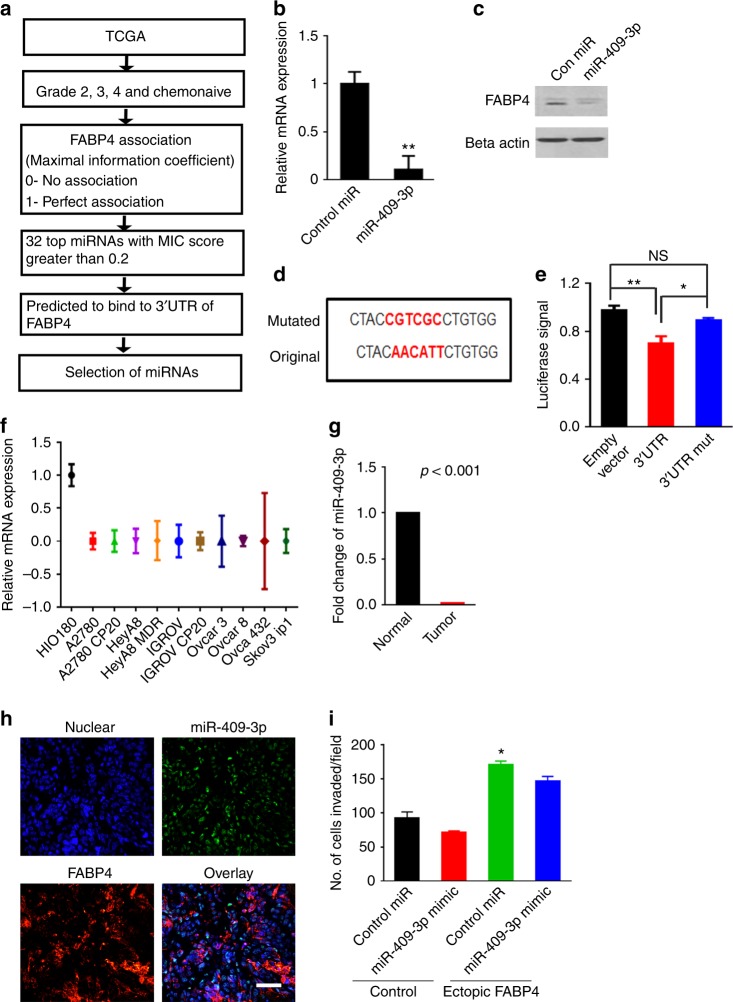
Upstream regulation of FABP4. **a** Flowchart showing the methods used to identify microRNAs (miRNAs) that target FABP4 in ovarian cancer. **b** Effect of miR-409-3p mimic transfection specifically on FABP4 expression levels (RNA) in HeyA8 MDR cells. **c** Effect of miR-409-3p mimic transfection on FABP4 expression levels (protein) in HeyA8 MDR cells. **d** Original and mutated 3’ untranslated region (UTR) sequence of FABP4, where miR-409-3p is predicted to bind. **e** Relative luciferase activity normalized to that of cells transfected with an empty control and cells transfected with control miRNA. HeyA8 MDR cells were transfected with empty control, wild-type FABP4 (red bar), or FABP4 with mutated 3’UTR (blue bar) and then transfected with a control miRNA mimic or a miR-409-3p mimic. Activity was measured 48 h after transfection. *p < 0.05; **p < 0.01; NS indicates not significant. **f** Expression of miR-409-3p in various ovarian cancer cell lines compared with normal ovarian cells (HIO180). **g** Expression of miR-409-3p in normal ovarian tissue and ovarian tumor samples in the GSE15190 dataset. **h** In situ hybridization for miR-409-3p (green) and FABP4 (red) in tumor tissues from patients with ovarian cancer (*n* = 8). Representative image is shown. **i** Invasion potential of SKOV3 ip1 cells was assessed in control cells or cells ectopically expressing FABP4 after transfection with control miRNA mimic or miR-409-3p mimic. Bars and errors bars represent the means and the corresponding SEMs for *n* > 3

Next, we investigated the potential mechanisms by which FABP4 is regulated in tumors. We first sought to determine whether there is any association between copy number and mRNA expression of *FABP4*. We examined The Cancer Genome Atlas (TCGA) datasets across platforms (Affymetrix, Agilent, RNASeqv2) and found no correlation between copy number and mRNA expression (Supplementary Fig. 3a). We also examined whether microRNAs (miRNAs) could regulate FABP4 levels. Our miRNA selection process is presented in Fig. 2a. We probed the TCGA dataset and selected samples from chemotherapy-naive cases of high-grade serous ovarian cancer. In these samples, we looked for miRNAs that were associated with FABP4 levels. To narrow our search further, we used the maximum information coefficient score^17^, where a score of 0 indicates no association and a score of 1 indicates perfect association between a gene and a miRNA. Thirty-two miRNAs with a score >0.2 were selected (Supplementary Table 1). We then selected miRNAs from this group that were predicted to bind to the 3′ untranslated region (UTR) of FABP4 by miRNA–gene interaction prediction software. Mir-409-3p was predicted to target FABP4 by nine different prediction software programs.

Next, we transfected HeyA8 MDR cells with miR-409-3p mimic, which led to a significant decrease (85%, *p* < 0.01) in FABP4 expression (Fig. 2b). This effect was also observed at the protein level (Fig. 2c). Similar effect was observed in another cell line (Ovcar 5, *p* < 0.01, Supplementary Fig. 3b; Ovca 432, *p* < 0.05, Supplementary Fig. 3c). We also transfected ovarian cancer cells (Ovcar 3) with anti-miR-409-3p and then examined *FABP4* expression. Inhibition of miR-409-3p led to a significant increase in *FABP4* expression (*p* < 0.05; Supplementary Fig. 3d).

To determine whether miR-409-3p binds to the 3′UTR of *FABP4*, we conducted a luciferase assay. The predicted binding site for miR-409-3p on *FABP4* is presented in Supplementary Fig 3e. We also mutated the binding site; the original sequence and mutated sequences are shown in Fig. 2d. In cells treated with a miR-409-3p mimic, the relative luciferase activity was significantly lower for cells transfected with *FABP4* 3′UTR than in cells transfected with an empty control. When *FABP4*’s 3′UTR binding site was mutated, abrogation of binding activity was observed (Fig. 2e), suggesting that miR-409-3p binds to the 3′UTR of *FABP4*.

We next compared the expression of miR-409-3p in various ovarian cancer cell lines and non-transformed ovarian epithelial cells (HIO180). Compared with the control cells, all ovarian cancer cell lines showed low expression of miR-409-3p (Fig. 2f). We also analyzed the expression of FABP4 in these cell lines to determine the correlation between *FABP4* and miR-409-3p at the basal level (Supplementary Fig 3f) and observed an inverse association between miR-409-3p and *FABP4* in the cell lines. We then probed a Gene Expression Omnibus dataset (GSE15190)^18^ and compared the expression of miR-409-3p in serous ovarian tumor tissues with that of normal ovarian tissue samples. The dataset revealed that miR-409-3p is expressed at significantly lower levels in ovarian tumors than in normal ovarian samples (Fig. 2g). We also examined 25 patient samples for correlations between miR-409-3p and FABP4 expression. In situ hybridization staining revealed a clear inverse association in the staining pattern of FABP4 and miR-409-3p (Fig. 2h).

To investigate whether the functions of miR-409-3p are mediated through FABP4, we performed a rescue experiment where we stably expressed a *FABP4* construct lacking the 3′UTR in SKOV3ip1 cells so that it would not be sensitive to a miR-409-3p mimic. Compared to controls, SKOV3ip1 cells with ectopically expressed FABP4 were significantly more invasive (*p* < 0.05). Treatment of the cell line ectopically expressing FABP4 with miR-409-3p mimic did not result in a significant decrease in invasion (Fig. 2i), suggesting that FABP4 is an important target of miR-409-3p.

### Effect of miR-409-3p on tumor progression and FABP4 expression

**Fig. 3:**
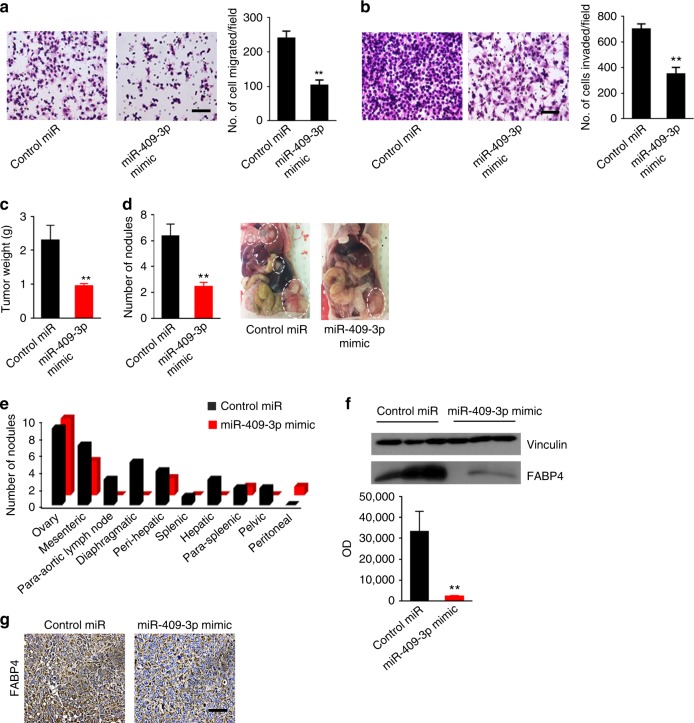
Effect of miR-409-3p on tumor progression and FABP4. **a**, **b** Effect of miR-409-3p mimic transfection on the **a** invasiveness and **b** migration of HeyA8 MDR cells. **p < 0.01. *n* = 3. **c** Aggregate tumor mass in orthotopic mouse models of ovarian cancer (HeyA8 MDR cell line) treated with control miRNA or miR-409-3p mimic incorporated in DOPC liposomes. **p < 0.01. *n* = 3. **d** Effect of miR-409-3p mimic on the number of nodules. Representative images are shown on the right. **p < 0.01. *n* = 3. **e** Distribution of tumor nodules in individual mice after treatment with control miRNA or miR-409-3p mimic. **f** Effect of treatment with miR-409-3p mimic on the expression of FABP4 (protein) in tumor samples (*n* = 3) Optical density (OD) is presented. **p < 0.01. **g** Representative images of immunohistochemical analysis of FABP4 expression in mice treated with control miRNA or miR-409-3p mimic. Bars and errors bars represent the means and the corresponding SEMs for *n* > 3

To determine whether miR-409-3p has tumor-suppressive properties, we transfected HeyA8 MDR cells with control miRNA or miR-409-3p mimic and conducted migration and invasion assays. Transfection of the miR-409-3p mimic led to a significant decrease in the migratory and invasive potential of cancer cells (*p* < 0.01; Fig. 3a, b). In contrast, reduction of miR-409-3p with anti-miR-409-3p resulted in increased migration (*p* < 0.05) and invasion of cancer cells (Supplementary Fig. 4a and b). The effect of miR-409-3p mimic transfection was also observed with the Ovcar 5 cells. Migration (Supplementary Fig 4c) and invasion (Supplementary Fig 4d) potential of Ovcar 5 cells were significantly reduced after miR-409-3p transfection (**p < 0.01).

To examine the effects of miR-409-3p in vivo, we used the HeyA8 MDR orthotopic mouse model. Starting seven days after cell injection, DOPC nanoliposomes containing control miRNA or miR-409-3p were administered intraperitoneally twice weekly. Compared with controls, mice treated with miR-409-3p mimic showed significant reduction in tumor weight (~60%, *p* < 0.01; Fig. 3c) and number of tumor nodules (~60%, *p* < 0.01; Fig. 3d). The treated mice also showed a different pattern of metastasis. Control mice had widespread metastasis in lymph nodes, diaphragm, pelvis, perisplenic, and perihepatic areas, whereas the incidence of metastasis was significantly lower in treated mice (Fig. 3e). Importantly, treatment with miR-409-3p mimic led to significant reduction of FABP4 protein (Fig. 3f, g) and RNA levels (*p* < 0.05; Supplementary Fig. 4e). There was no deleterious effect of the treatment on the mice weight (Supplementary Fig. 4f).

### Tumor microenvironmental factors in the regulation of miR-409-3p and FABP4

**Fig. 4:**
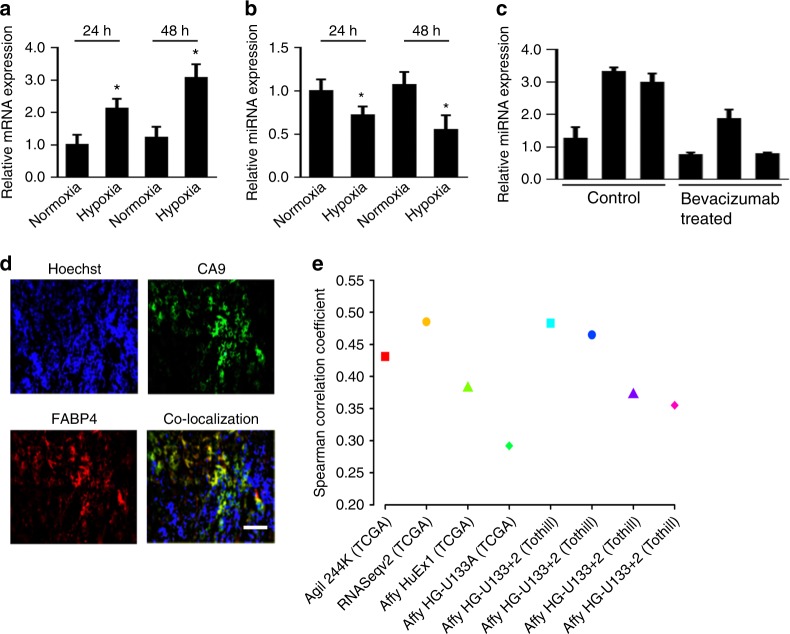
Tumor microenvironmental factors in the regulation of miR-409-3p and FABP4. **a**, **b** Effect of hypoxia on **a** FABP4 expression and **b** expression of miR-409-3p in HeyA8 MDR cells at 24 h and 48 h. **c** miR-409-3p expression levels in tumor tissues from mice treated with control vehicle or bevacizumab, which is known to induce hypoxia (*n* = 3 mice per group). **d** Co-localization of CA9 (a hypoxia marker) and FABP4 in tumor tissues taken from mice treated with bevacizumab, which is known to induce hypoxia. Representative image is shown. **e** Direct correlation of FABP4 with PLAU (a hypoxia signature gene) in patient samples across different platforms. Bars and error bars represent the means and the corresponding SEMs for *n* > 3

To identify the effects of the tumor microenvironment on FABP4 expression, we next examined various factors, including co-cultures with fibroblasts, macrophages, mesothelial cells and hypoxia treatment (Supplementary Fig. 5a). Of the factors examined, hypoxia (1% O_2_) consistently led to a significant increase in *FABP4* expression in cancer cells at 24 and 48 h (Fig. 4a). We also examined the expression of miR-409-3p after hypoxia treatment and observed low expression in hypoxic compared with normoxic cells at 24 and 48 h (Fig. 4b). We observed the same effect in an additional cell line (Ovca 432; Supplementary Fig. 5b and c); upon exposure to hypoxia, the expression of miR-409-3p was significantly reduced, whereas *FABP4* expression was significantly increased.

We then assessed the effect of hypoxia on miR409-3p expression in tumors treated with bevacizumab. This therapy, which targets vascular endothelial growth factor, has been shown to lead to hypoxia in tumors^19^. Hence, we compared the miR-409-3p expression between bevacizumab-treated and control tumors. Expression of miR-409-3p was lower in tumors from mice treated with bevacizumab compared to control tumors (Fig. 4c). We then stained the tumor sections taken from these mice with CA9 (a hypoxia marker) and FABP4; we observed co-localization of FABP4 with CA9 (Fig. 4d). We further analyzed the TCGA and Tothill ovarian cancer datasets and observed a significant positive correlation between FABP4 and PLAU (a gene from hypoxia metagene signature) across all platforms (Fig. 4e)^20^. The correlations of *FABP4* with other genes from the metagene signature are presented in Supplementary Table 2.

### Effect of FABP4 expression on protein expressions in cancer cells

**Fig. 5:**
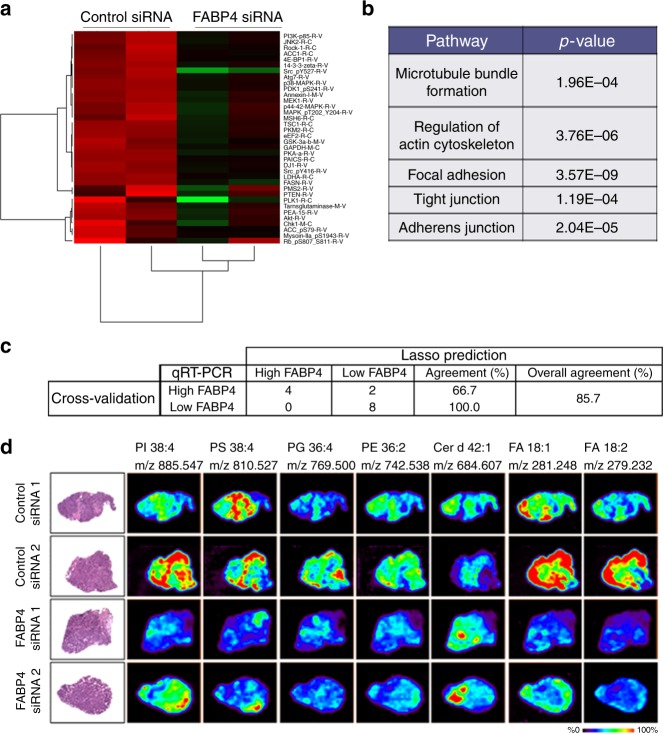
Downstream effect of FABP4. **a** RPPA data for downregulated proteins in the *FABP4* siRNA and control siRNA groups. **b** Netwalker analysis of RPPA data. **c** Model correlating molecular profiles and FABP4 expression based on the combined (negative and positive) ion mode data. **d** DESI-MS images of in vivo tumor samples from control siRNA and *FABP4* siRNA treatment groups

To identify signaling pathways downstream of FABP4, we conducted reverse phase protein arrays (RPPA) after silencing FABP4 in HeyA8 MDR cells. The heat map of the RPPA data (Fig. 5a) showed that several proteins (Supplementary Fig. 6a and Supplementary Data 1) were downregulated after FABP4 knockdown. We further used Netwalker software to analyze the protein data, and the results showed sub pathways pertaining to metastasis being significantly downregulated in the *FABP4* siRNA group (Fig. 5b).

### Effect of FABP4 expression on ovarian cancer tissue metabolites

To explore the effects of FABP4 on metabolic changes in human ovarian cancer tissues, we first analyzed previously published gene expression and metabolomics data of high grade serous ovarian carcinoma tissues^21–23^. From those results, several metabolites were specifically expressed in either low or high FABP4 expressing patient tumor samples (Supplementary Table 3). Next, we evaluated metabolic and lipid trends related to *FABP4* gene expression by desorption electrospray ionization mass spectrometry imaging (DESI-MSI). In this study, a total of 31 high-grade ovarian cancer tissue samples were analyzed by DESI-MSI in both negative and positive ion modes to investigate a broad range of lipid and metabolites related to FABP4 expression. Using this approach, only the data specific to the tumor region were selected and evaluated, thus eliminating the inclusion of stromal lipids and metabolites.

SAM analysis (Significance Analysis of Microarrays) was conducted to identify statistically significant *m/z* values (mass to charge ratios) detected by DESI-MSI characteristic of low- or high-FABP4 expression. Positive SAM scores represent higher relative abundance in samples with low-FABP4-expression, and negative SAM scores represent higher relative abundance in samples with high-FABP4-expression. Negative ion mode data revealed 361 and 289 monoisotopic *m/z* values associated with low or high FABP4 expression, respectively, with false discovery rate (FDR) of <3.8%. From these, 238 *m/z* values were tentatively identified by high mass accuracy measurements and tandem MS analysis (Supplementary Table 4) as small metabolites, fatty acids (FA), ceramides (Cer), glycerolipids (GL), monoacylglycerophosphates (PA), glycerophosphoethanolamines (PE), glycerophosphoglycerols (PG), glycerophosphoinositols (PI), glycerophosphoserines (PS), and cardiolipins (CL). Molecular formulas, detected *m/z* and mass errors in ppm are provided in Supplementary Table 5 for the species identified. Fatty acid and metabolite composition was observed to significantly differ between the two expression groups. For example, higher unsaturation and oxidation of fatty acid species was selected as characteristic of high FABP4 expression. A significant number of *m/z* values related to high FABP4 expression corresponded to GL, PE, PG, and lysophospholipid species, such as LysoPE, LysoPG, and LysoPI. On the other hand, CL species were almost exclusively selected with the low-FABP4-expression type. These variations in lipid composition suggest alterations in metabolism and biosynthesis due to the changes in the expression of the FABP4 gene.

We then applied the Lasso (Least absolute shrinkage and selector operator)^24^ method to evaluate if predictive statistical models could be built to classify samples as having low- or high-FABP4 expression based on metabolite composition. The samples were randomly divided into a training and validation set of samples (50:50). Results based on negative ion mode data yielded an overall agreement of 81.6% and 0.79 area under the curve (AUC) value for the training set performance. The remaining independent set of samples were classified with an overall agreement of 56.1% and AUC value of 0.60 (Table 1). In positive ion mode data, an overall agreement of 74.2% for CV (AUC = 0.73) and 61.7% agreement for the validation set (AUC = 0.58) (Table 1) were achieved. To improve prediction of FABP4 expression, positive and negative ion mode data were combined to build a comprehensive model. Using this approach, 66.7% of high-FABP4 patients and 100% of low-FABP4 patients were successfully classified in patient-by-patient analysis (Fig. 5c and Table 1).

**Table 1:** Lasso classification of high grade serous cancer samples into high and low FABP4 expression based on DESI-MS lipid and metabolite data in negative, positive and combined polarities

				**Lasso prediction**				
			**qRT-PCR**	**High FABP4**	**Low FABP4**	**Agreement (%)**	**Overall agreement (%)**	**AUC**
Negative ion mode	For pixels	Cross-validation	High FABP4	1925	671	74.2	81.6	0.79
			Low FABP4	1218	6450	84.1		
		Validation set	High FABP4	1003	1189	45.8	56.1	0.6
			Low FABP4	1084	1906	63.8		
	For patients	Cross-validation	High FABP4	5	1	83.3	80	0.79
			Low FABP4	3	11	78.6		
		Validation set	High FABP4	4	2	66.7	63.6	0.6
			Low FABP4	2	3	60		
Positive ion mode	For pixels	Cross-validation	High FABP4	1248	531	70.2	74.2	0.73
			Low FABP4	2271	6823	75		
		Validation set	High FABP4	661	705	48.4	61.7	0.58
			Low FABP4	1049	2169	67.4		
	For patients	Cross-validation	High FABP4	5	1	83.3	73.7	0.73
			Low FABP4	4	9	69.2		
		Validation set	High FABP4	2	2	50	50	0.58
			Low FABP4	3	3	50		
Combined polarities	For patients	Cross-validation	High FABP4	4	2	66.7	85.7	-
			Low FABP4	0	8	100		

Results are shown for pixels and patient classification both for the training set (cross-validation) and validation set. Agreement is calculated comparing lasso prediction to FABP4 expression provided by qRT-PCR. *AUC* area under the curve.

To further investigate direct changes in lipid and metabolite levels due to the changes in FABP4 expression, we analyzed in vivo tissues from high-grade ovarian cancer mouse models presented in Fig. 1i–l. Using DESI-MSI, we compared tumor tissues of *FABP4* siRNA group (low FABP4 expression, *n* = 3) to control siRNA samples (high FABP4 expression, *n* = 3). SAM analysis revealed 627 monoisotopic *m/z* values characteristic of FABP4 silencing, with FDR < 5.5%. From the selected *m/z* values, a total of 184 species (59 increased in low FABP4, 125 increased in high FABP4) were identified as metabolites, FA and complex lipids, with attributed SAM scores associated to FABP4 expression (Supplementary Table 6). Overall, the majority of the lipid classes received negative SAM scores, thus being associated to high FABP4 expression. For example, FA and monoacylglycerophosphates (PA) were selected exclusively for characterization of the control siRNA samples. On the other hand, Cer species were associated with low expression of the *FABP4* gene. These trends were also reflected by the relative abundances observed in the ion images as shown in Fig. 5d, where all the lipid species shown except for the Cer d42:1 displayed higher relative abundances within the high FABP4 samples. Remarkably, comparison between the metabolic species selected by SAM analysis for both human and mouse samples resulted in a total of 76 common *m/z* values related to high or low FABP4 expression (Table 2, Supplementary Table 7). Among these, various FA species were associated with high expression of the *FABP4* gene in both the mice and human samples. Interestingly, common GL and PI species for both animal samples were specific to the high FABP4 class, while Cer species were characteristic of tissues with low FABP4 expression. Differences in FA chain length and saturation level were also observed within the same GP class. For example, longer FA chain in PE and lysoPE species were associated with high FABP4 expression, while longer FA chain in PS lipids were characteristic to tissue samples with lower FABP4 expression. These similar metabolic trends observed in both human and mice samples strongly suggest and corroborate the effects of the *FABP4* gene in metabolism and biosynthesis.

**Table 2:** Lipid and metabolite species related to low and high expression of the FABP4 gene in both human and mice samples based on SAM analysis from DESI-MSI data

Metabolic species selected by SAM in both human and mice samples					
**siFABP4–low FABP4 expression**		**Control–high FABP4 expression**			
Attribution	Molecular formula	Attribution	Molecular formula	Attribution	Molecular formula
Metabolites		Metabolites		Fatty acids	
Succinate	C_4_H_5_O_4_	Taurine	C_2_H_6_NO_3_S	FA 18:3	C_18_H_29_O_2_
Glutathione	C_10_H_16_N_3_O_6_S	Xanthine	C_5_H_3_O_2_N_4_	FA 18:2	C_18_H_31_O_2_
Glycerophosphoethanolamines		Glycerophosphoethanolamines		FA 18:1	C_18_H_33_O_2_
LysoPE 18:1	C_24_H_45_NO_8_P	LysoPE 16:0	C_21_H_43_NO_6_P	FA 18:0	C_18_H_35_O_2_
PE 18:1/18:1	C_41_H_77_NO_8_P	LysoPE 18:0	C_23_H_47_NO_7_P	FA 19:0	C_19_H_37_O_2_
PE 22:6/16:0	C_43_H_73_NO_8_P	PE P-18:0/18:4	C_41_H_73_NO_7_P	FA 20:5	C_20_H_29_O_2_
Glycerophosphoglycerols		PE P-38:4	C_43_H_77_NO_7_P	FA 20:4	C_20_H_31_O_2_
PG 18:0/18:1	C_42_H_80_O_10_P	PE 20:4/18:1	C_43_H_75_NO_8_P	FA 18:2	C_18_H_32_O_2_Cl
PG 18:0/18:0	C_42_H_82_O_10_P	PE 40:5	C_45_H_79_NO_8_P	FA 18:1	C_18_H_34_O_2_Cl
PG 42:7	C_48_H_80_O_10_P	PE 22:4/18:0	C_45_H_81_NO_8_P	FA hydroxy 20:4	C_20_H_31_O_3_
Ceramides		Glycerophosphoglycerols		FA 22:6	C_22_H_31_O_2_
Cer d18/16:0	C_34_H_69_NO_3_Cl	LysoPG 18:2	C_24_H_44_O_9_P	FA 22:5	C_22_H_33_O_2_
Cer m18:1/22:0	C_40_H_79_NO_2_Cl	LysoPG 18:1	C_24_H_46_O_9_P	FA 22:4	C_22_H_35_O_2_
Cer m42:1	C_42_H_83_NO_2_Cl	LysoPG 22:6	C_28_H_44_O_9_P	FA 22:3	C_22_H_37_O_2_
Cer d42:1	C_42_H_83_NO_3_Cl	PG 16:0/18:1	C_40_H_76_O_10_P	FA 20:4	C_20_H_32_O_2_Cl
Cer d42:0	C_42_H_85_NO_3_Cl	PG 18:2/18:2	C_42_H_74_O_10_P	FA 24:5	C_24_H_37_O_2_
Cer d18:1/26:1	C_44_H_85_NO_3_Cl	PG 18:2/18:1	C_42_H_76_O_10_P	FA 24:4	C_24_H_39_O_2_
Cer d18:1/26:0	C_44_H_87_NO_3_Cl	PG 18:1/18:1	C_42_H_78_O_10_P	FA 22:4	C_22_H_36_O_2_Cl
Cardiolipins		PG 20:4/20:4	C_46_H_74_O_10_P	Glycerophosphoinositols	
CL 70:7	C_79_H_138_O_17_P_2_	Cardiolipins		LysoPI 18:0	C_27_H_52_O_12_P
CL 70:6	C_79_H_140_O_17_P_2_	CL 72:4	C_81_H_148_O_17_P_2_	LysoPI 20:4	C_29_H_48_O_12_P
CL 74:10	C_83_H_140_O_17_P_2_	Glycerolipids		PI 20:3/17:1	C_46_H_80_O_13_P
CL 74:9	C_83_H_142_O_17_P_2_	MG 18:0/0:0	C_21_H_40_O_4_Cl	PI 18:1/20:4	C_47_H_80_O_13_P
Glycerophosphoserines		DG 36:3/0:0	C_39_H_70_O_5_Cl	PI 18:0/20:4	C_47_H_82_O_13_P
PS P-36:2	C_42_H_77_NO_9_P	DG 36:2/0:0	C_39_H_72_O_5_Cl	PI 18:0/20:3	C_47_H_84_O_13_P
PS P-36:1	C_42_H_79_NO_9_P	Glycerophosphoserines		PI 18:0/22:6	C_49_H_82_O_13_P
PS 18:0/18:1	C_42_H_79_NO_10_P	PS 18:1/18:2	C_42_H_75_NO_10_P	PI 18:0/22:4	C_49_H_86_O_13_P
PS 40:2	C_46_H_85_O_10_NP	PS 18:0/20:4	C_44_H_77_O_10_NP		
PS 40:1	C_46_H_87_O_10_NP	PS 39:4	C_45_H_79_NO_10_P		
PS 18:1/24:1	C_48_H_89_O_10_NP				
PS 18:1/24:0	C_48_H_91_O_10_NP				

### Effect of FABP4 on patient survival

**Fig. 6:**
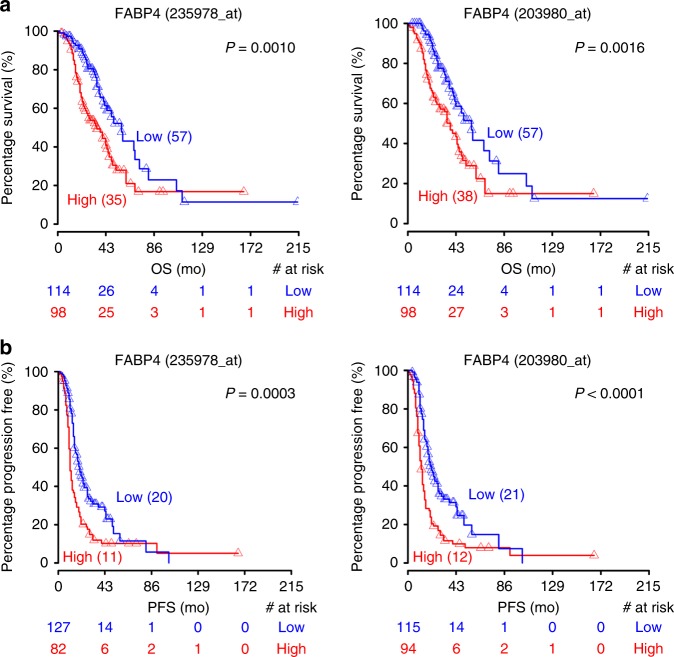
Effect of FABP4 on patient survival. **a**, **b** Kaplan–Meier plots for overall (**a**) and progression-free (**b**) survival in ovarian cancer patients, based on FABP4 expression for both the probes associated with FABP4. The data were extracted from Tothill data set

High FABP4 expression was significantly correlated with poor overall (Fig. 6a) and progression-free (Fig. 6b) survival (*p* < 0.01) in the Tothill dataset^25^ for both FABP4 probe sets. The multivariate analysis for overall (Supplementary Fig 6b) and progression-free (Supplementary Fig 6c) survival as well as the univariate analysis for overall (Supplementary Fig 6d) and progression-free (Supplementary Fig 6e) survival are presented.

### Effect of tamoxifen on FABP4 expression and function

Having established the importance of FABP4 as a candidate target for ovarian cancer treatment, we next wished to identify clinically approved drugs that could be used to inhibit FABP4. Tamoxifen, a selective estrogen receptor modulator, has been shown to inhibit FABP4 in macrophages^26^. We checked whether it can be used to target FABP4 in ovarian cancer cells. We used physiologically relevant concentrations of tamoxifen to determine its effect on FABP4 expression and function in ovarian cancer cells. At a concentration of 3.5 µM, tamoxifen decreased the expression of *FABP4* by approximately 50% (*p* < 0.05; Supplementary Fig. 7a). As a result of this downregulation, the ability of cancer cells to take up free fatty acids was also impaired. The cells treated with tamoxifen exhibited lower uptake compared to untreated control cells (*p* < 0.01; Supplementary Fig. 7b). The treatment also affected the migratory potential of cancer cells and resulted in a significant decrease in the number of migrated cells (Supplementary Fig. 7c). The effect of tamoxifen on *FABP4* was also observed in A2780 cells where tamoxifen treatment significantly downregulated *FABP4* expression (Supplementary Fig 7d) as well as the ability of cells to migrate (Supplementary Fig 7e) (**p < 0.01).

## Discussion

Systematically coupling bioinformatics, experimental models and clinical data, we elucidated aspects of the underlying biology of ovarian cancer that could lead to aggressive metastasis of ovarian cancer. High expression of FABP4 is a known predictor of residual disease^4^. Our preclinical studies show that ectopic expression of FABP4 increases tumor progression while in vivo experiments with DOPC nanoliposomes show that silencing FABP4 reduces the extent of metastasis. Our findings thus provide a promising therapeutic strategy that can potentially be useful for patients at high risk of being left with residual disease at initial surgery. Previous studies have shown that FABP4 present in the stromal cells can lead to cancer progression by providing a source of energy to cancer cells or increasing angiogenesis^12,14^. However, our study presents cancer cell specific FABP4 as a crucial player in promoting invasive and infiltrative metastasis in ovarian cancer. Metabolic changes in ovarian cancer have been studied before^27,28^. However, without spatial information, it is difficult to determine whether the metabolic alterations are occurring in the tumor or stromal compartment. To focus on the tumor compartment, we employed the DESI-MS imaging technique which allowed us to identify the chemical species present in the tissue as well as their spatial distribution. Analysis of the data, specific to tumor compartment of patient samples revealed that a unique metabolic profile is associated with the expression of FABP4. Higher unsaturation and oxidation of fatty acids and lysophospholipids were observed in higher relative abundance in samples with higher FABP4 expression. Several studies have suggested that unsaturated fatty acids play a key role in pathways leading to tumor progression by activating the beta catenin pathway, downregulating PTEN or increasing cancer cell adhesion^29–31^. Fatty acid oxidation has also been shown to increase metastasis in breast and ovarian cancer models^14,32^. Lysophospholipids have also been known to stimulate cancer cell migration and they are also considered as potential biomarkers for ovarian cancer^33,34^. High expression of FABP4 can thus regulate various metabolites and protein pathways that can lead to aggressive metastasis of ovarian cancer. This data elucidating the key metabolic determinants of ovarian cancer metastasis thus lay a strong foundation for future studies which can focus on the development of therapeutic strategies targeting these species.

Previous studies have identified PPARγ and endothelial DLL4-NOTCH as regulatory factors for FABP4^11,35,36^. Our findings provide a new understanding of FABP4 regulation by miR-409-3p. MiR-409-3p has been known to target several genes such as MGMT, HMGN5 (glioblastoma)^37,38^, beclin, NLK and GAB1 (Colon cancer)^39–41^, AKT, ZEB1 (Breast cancer)^42,43^, c-Met (Lung cancer)^44^, radixin, PHF10 (gastric cancer)^45,46^. In these cancer types, miR-409-3p has been known to act as a tumor suppressor whereas in prostate cancer miR-409-3p has been shown to increase EMT and stemness markers in cancer cells^47^. The role of miR-409-3p in ovarian cancer, however, was not known. Here, we demonstrate that miR-409-3p acts as an effective tumor suppressor in ovarian cancer through its effects on FABP4. The antitumor properties of miR-409-3p stem from its inhibitory effects on FABP4, as shown by the fact that restoration of FABP4 rescued the tumor suppressive effects of miR-409-3p on cancer cells. Importantly, we show that the DOPC nanoliposomes containing a miR-409-3p mimic effectively reduce tumor burden and metastasis in vivo. Hypoxia has been linked with ovarian cancer progression^48,49^. However, the exact role of hypoxia in tumor metastatic patterns was not well understood. We found that hypoxia can downregulate miR-409-3p, thus potentially removing its inhibitory effect on FABP4, which subsequently results in increased FABP4 levels. Recent findings have also suggested that HIF-1α can directly bind to the promoter region of FABP4^50^. The cross-talk between these regulators of FABP4 and their specific place and importance in the FABP4 pathway presents an interesting avenue for future studies.

Previous studies have shown that increased fatty acid uptake by the cells lead to increased motility and EMT^51,52^. Since FABP4 is a key molecule involved in free fatty acid uptake, inhibiting its ability to uptake fatty acid could be a potential therapeutic avenue to inhibit cancer metastasis. We thus identified tamoxifen as a potential drug of interest that could inhibit FABP4 and affect migration of ovarian cancer cells. Although, additional research is warranted, our data suggest tamoxifen as a promising drug for high grade serous ovarian cancer treatment, possibly in a maintenance setting.

**Fig. 7:**
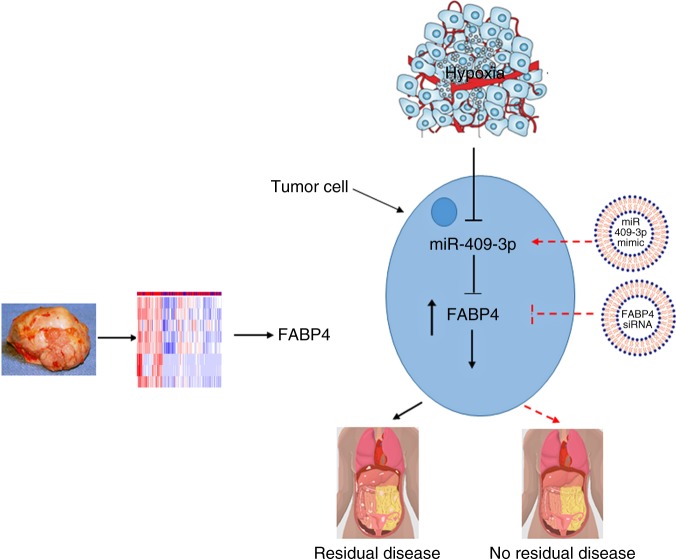
Summary model. Schematic representation of regulation of FABP4, potential therapeutic strategies to target FABP4 and miR-409-3p and downstream pathological consequences of manipulating their expression

In summary, our study provides a new understanding of a previously unrecognized mechanism (Fig. 7) by which FABP4 leads to altered tumor metastasis patterns and a higher extent of disease in ovarian cancer. Collectively, our data offer candidate targets for therapeutic interventions for ovarian cancer patients.

## Methods

### Cell line maintenance and siRNA and miRNA transfection

All cancer cell lines were maintained at 37 °C and 5% CO_2_ in culture with RPMI-1640 or Dulbecco’s modified Eagle medium supplemented with 15% fetal bovine serum and 0.1% gentamicin sulfate (Gemini Bio-Products, Calabasas, CA, USA). HeyA8MDR, A2780, SKOV3ip1, Ovcar 5, and Ovcar 3 were received from the MDA Characterized Cell Line Core (CCLC) or directly from ATCC. Ovca 432 was received from the originator, Dr. Ronny Drapkin.

All siRNA transfections were conducted using Lipofectamine2000 as a transfecting agent. SiRNA concentration of 100 nM was used, and the ratio of Lipofectamine (Life Technologies, Carlsbad, CA, USA) to a specific siRNA was 3:1. The cells were treated with siRNAs for 4 h in serum-free media before incubation in complete media for the specified time frame. For miRNA transfections (mimic and anti-miRNA), RNAiMAX (Life Technologies, Carlsbad, CA, USA was used as a transfection agent, and the ratio of RNAiMAX to a specific miRNA was 2:1. The concentration of miRNA-mimic or anti-miR inhibitor was 40 nM concentration and the transfections were conducted in serum-free conditions. After 4 h, the cells were incubated in complete media for the specified time frame. The sequences for siRNAs and miRNAs are listed in Supplementary Table 8.

### In vivo models

Female athymic nude mice were purchased from Taconic Farms (Hudson, NY) and housed in pathogen-free conditions. The mice were cared for according to the guidelines of the American Association for Accreditation for Laboratory Animal Care International and the US Public Health Service Policy on Humane Care and Use of Laboratory Animals. All in vivo experiments and protocols were approved by MD Anderson’s Institutional Animal Care and Use Committee.

To establish the tumors, 1 × 10^6^ cells/mouse A2780 cells, 8 × 10^5^ cells/mouse HeyA8 MDR-Luc cells, or 1 × 10^6^ cells/mouse Ovcar 5-Luc cells were injected into the ovary. No therapeutic intervention was conducted in the experiment in which FABP4-overexpressing cells were injected. For all therapeutic experiments, a siRNA or miRNA dose of 200 μg kg^−1^ was used, and the treatments were started 1 week after cell injections. The mice were divided into two groups: control and treatment, 10 mice/group. Mice in the control group received control siRNA or control miRNA incorporated into neutral DOPC liposomes. Mice in the treatment group received *FABP4* siRNA or a miR-409-3p mimic incorporated into DOPC liposomes. The doses were given twice weekly intraperitoneally. The mice were monitored daily for any toxic effects. Luciferase imaging was conducted to observe the effect of treatments on the metastasis as described previously^53^. Briefly, imaging and data acquisition were performed using the IVIS Spectrum in vivo imaging system coupled to Living Image Software (Xenogen). The mice were first anesthetized in an acrylic chamber with a mixture of 1% isoflurane. They were then injected intraperitoneally with luciferin potassium salt (15 mg ml^−1^) in PBS at a dose of 150 mg kg^−1^ body weight. A digital grayscale image was initially acquired, which was then overlaid with a pseudocolor image representing the spatial distribution of detected photons emerging from active luciferase present within the animal. Signal intensity was expressed as a sum of all photons detected per second. Once a mouse in any group became moribund, all mice were euthanized. Mouse weight, tumor weight, number of nodules, and locations of metastasis were recorded. Tumor tissues were then frozen in optimal cutting temperature media, fixed in formalin for paraffin embedding, or snap-frozen.

### Liposomal nanoparticle preparation

Incorporation of siRNA or miRNA into DOPC liposomes was achieved as previously described^54^. Briefly, DOPC and siRNA or miRNA were mixed at a ratio of 1:10 (w/w) in the presence of tertiary butanol. Tween 20 was added to the mixture at a ratio of 1:19. The mixture was vortexed and frozen in an acetone/dry ice bath and lyophilized. The lyophilized preparations were hydrated with PBS at room temperature to a concentration of 200 µg of siRNA or miRNA kg^−1^ per injection per mouse.

### MiRNA–mRNA expression association in TCGA OV samples

TCGA cases were included if the patients had high-grade disease and no history of pre-treatment. TCGA gene expression quantification were produced using justRMA applied to the CEL files from TCGA (Affymetrix HT HG-U133A arrays, *n* = 598)^4^. TCGA miRNA microarray level 3 data (Agilent 8 × 15 K Human miRNA-specific microarray) was obtained from the Data Access Matrix. Data was available for 799 miRNAs. There were 541 samples meeting the inclusion criteria which had both gene expression and miRNA data available. The association between each probe for the gene of interest and each available miRNA was assessed using the Maximal Information Coefficient (MIC) computed using the publicly available MINE software of Reshef et al^17^.

### MiRNA–mRNA Interactions

We retrieved miRNA–target interaction information from miRWalk2.0 (http://www.umm.uni-heidelberg.de/apps/zmf/mirwalk/) that hosts miRNA–target predictions from twelve programs. We selected the cases predicted by at least seven algorithms (half of the total number of programs checked + 1). The interaction between miR-409-3p and FABP4 (NM_001442) (3′UTR) was predicted by nine programs (miRWalk, Microt4, miRanda, miRMap, miRNAMap, PITA, RNA22, RNAhybrid, Targetscan). We used Perl to sort the information available and Latex to present the sites most probable to interact.

### Luciferase reporter assays, and *FABP4* 3′UTR site mutagenesis

Luciferase assays were conducted as described previously^55^. Briefly, a GoClone pLightSwitch luciferase reporter for the 3′UTR of *FABP4* was purchased from Switchgear Genomics (Menlo Park, CA, USA). HeyA8 MDR cells were transfected with control miRNA or miR-409-3p mimic (100 nM; Life Technologies, Carlsbad, CA, USA) with the help of FuGENE HD transfection agent. The cells were also transfected with 3′UTR reporter constructs and Cypridina TK controls (pTK-Cluc). After 24 h, the LightSwitch Dual Luciferase assay kit was used and the luciferase signal was measured using a microplate luminometer, as per the manufacturer’s guidelines (Biotek, Winooski, VT, USA). Luciferase activity was normalized using the Cypridina TK control, and an empty 3′UTR construct was used as a negative control. The ratios were then normalized to the scrambled control miRNA. Mutant *FABP4* 3′UTR was created for the predicted binding site mentioned in Fig. 2e using a QuickChange lightning multi-site-directed mutagenesis kit (Agilent Technologies, Santa Clara, CA, USA) using the primers mentioned in Supplementary Table 8 [AKS1] [PS2]. [SK3] The mutation was then confirmed using Sanger DNA sequencing before the mutant *FABP4* 3′UTR was used for the luciferase assay.

### Immunoblotting

Protein lysates from tumor tissues or cultured cells were prepared using modified RIPA buffer containing proteinase and phosphatase inhibitors. Protein concentrations were determined using the BCA protein assay reagent kit (Pierce Biotechnology, Rockford, IL, USA). Lysates were loaded and separated on SDS-polyacrylamide gels. The proteins were then transferred to a nitrocellulose membrane. The membrane was blocked at room temperature for 1 h in 5% milk powder in Tris-buffered saline with Tween 20 (TBST) and then incubated at 4 °C overnight with primary antibodies: FABP4 (catalog number HPA002188; SigmaAldrich) and vinculin (catalog number V9131; Sigma) or beta-actin (catalog number A5316, Sigma) as loading control. After washing with TBST, the membranes were incubated with horseradish peroxidase-conjugated donkey anti-rabbit IgG (for FABP4) or sheep anti-mouse IgG (for vinculin) (catalog numbers NA934 and NA931; 1:2000; GE Healthcare) at room temperature for 2 h. An enhanced chemiluminescence detection kit (catalog number NEL104001EA; Pierce Biotechnology) was used to visualize the horseradish peroxidase signal. Full blots are included in Supplementary Fig. 8.

### Migration and invasion assay

Corning Transwell inserts (Costar, MA, USA) were coated with 0.1% gelatin (migration) or defined basement membrane matrix (invasion). Defined basement membrane matrix (for invasion) was prepared in a 10 ml stock solution with laminin (50 µg ml^−1^), 1 ml of type IV collagen (50 µg ml^−1^), 0.2 ml of gelatin (2 mg ml^−1^), and 4 ml and 4.8 ml of PBS. In the upper chamber, HeyA8 MDR and Ovcar 5 cells (0.7 × 10^5^) suspended in 200 μl of serum-free media were added 48 h after siRNA/miRNA transfections or tamoxifen treatments. In the lower chamber, complete media for cells containing 10% fetal bovine serum (500 μl) was added as a chemo-attractant. The chambers were incubated at 37 °C in 5% CO_2_ for 6 h (migration assay) or 24 h (invasion assay). After incubation, the cells in the upper chamber were removed with cotton swabs. Cells were fixed, stained, and counted using light microscopy. Cells from five random fields were counted. Experiments were done in triplicate.

### Tumor samples

High-grade ovarian tumor samples were obtained from the Cooperative Human Tissue Network (CHTN) and MD Anderson Tissue Bank under an approved IRB protocol and written consent was obtained for the use of patient samples for research. The details regarding the quality control for the samples obtained from CHTN can be found at https://www.chtn.org/quality.html

### miR-409-3p expression in patient tumor samples as taken from GEO dataset

The detailed method can be found in Wyaman et al, 2009^18^. Briefly, 4 samples of HOSE (normal primary ovarian surface epithelial cells taken from postmenopausal women) and 19 serous epithelial ovarian cancer samples were analyzed. The tumor samples were high-grade and contained more than 70% malignant epithelial cellularity. The cloning frequency of a specific miR expressed as a fraction of total reads from a given sample was used to compare the relative expression of miRNAs between samples.

### In situ hybridization

In situ hybridization was done as previously described^55^. High grade serous ovarian cancer tissues which were chemonaïve were selected. Xylene and an ethanol dilution series were used for deparaffinization and rehydration of the formalin-fixed, paraffin-embedded tissue sections. Tissue sections were digested with 15 µg ml^−1^ proteinase K for 20 min at room temperature and then loaded onto Ventana Discovery Ultra (Tucson, AZ) for in situ hybridization analysis. The tissue slides were then incubated with a double-digoxigenin-labeled miRCURY LNA miRNA probe (Exiqon, Woburn, MA, USA) for 2 h at 55 °C. Three percent H_2_O_2_ was used to inactivate endogenous peroxidases. After incubation with polyclonal anti-digoxigenin antibody and horseradish peroxidase-conjugated secondary antibody (Ventana), a tyramine-conjugated fluorochrome (TSA) reaction was performed for 12 min. Sequential TSA rounds were performed for the detection of proteins using the same protocol. Slides were mounted with antifading ProLong Gold Solution (Life Technologies).

### Immunostaining

Paraffin-embedded sections (5 µm) of tumor tissues were cut and used for detection of FABP4. Formalin-fixed sections were deparaffinized by sequential washings with xylene, 100% ethanol, 95% ethanol, 80% ethanol, and PBS. After antigen retrieval, endogenous peroxidase was used to block the slides. Nonspecific binding was prevented by incubating the slides with 4% fish gelatin. This was followed by incubation with primary antibody overnight at 4 °C. The next day, the slides were washed with PBS and incubated with suitable secondary antibody (goat anti-rabbit horseradish peroxidase antibody diluted in protein block solution [JB4]) for 1 h at room temperature. The slides were then washed with PBS followed by development with 3,3′-diaminobenzidine. The nuclei were stained with Gill’s hematoxylin solution and the slides were then mounted.

For florescence staining, optimal cutting temperature-embedded frozen tissue samples were used. Nonspecific protein blocking was achieved using 4% fish-gelatin solution in tris-buffered saline with tween-20. Slides were incubated with primary antibody (CA9 or FABP4) overnight at 4 °C. For immunofluorescence, Alexa 594 or Alexa 499 were used for secondary antibody staining. Nuclear staining was performed using Hoechst 33342 (Molecular probes).

### Quantitative real-time PCR

Total RNA was extracted from the tumor tissues using the Direct-Zol RNA extraction kit (Zymo Research, Irvine, CA, USA). RNA was then quantified using a NanoDrop spectrophotometer method and the 260 nm/280 nm ratios were checked to determine quality. RNA (1 µg per sample) was reverse-transcribed into cDNA using the Verso cDNA kit (Thermo Scientific, West Palm Beach, FL, USA) according to the manufacturer’s protocol.

Quantitative real-time PCR was performed on an ABI7500 PCR system (Applied Biosystems, Warrington, UK) using 1 µl of cDNA for each sample. SYBR green (Applied Biosystems) was used to detect the products, and 20 pmol of primer was used for the reaction. All reactions were carried out with 20 µl of reaction mix and performed in triplicate. We used the following primers: for FABP4, 5′-TGATGATCATGTTAGGTTTGGC-3′ (forward) and 5′-TGGAAACTTGTCTCCAGTGAA-3′ (reverse); for 18 S, 5′CGCCGCTAGAGGTGAAATTC3′ (forward) and 5′TTGGCAAATGCTTTCGCTC3′ (reverse). The following conditions were used for PCR: 50 °C for 2 min, then 95 °C for 15 min, followed by 40 cycles at 95 °C for 1 min each. All reactions were analyzed using the ABI7500 Applied Biosystems PCR software (v.2.0.5). The cycle threshold values of the target genes were initially normalized to the cycle threshold values of 18 S rRNA, and melt curves were checked to determine the specificity of the reactions.

For miRNA quantifications, Taqman miRNA assays (Life Technologies) were used and reverse-transcription real-time PCR was performed, according to the manufacturer’s instructions. RNU6B was used as a housekeeping gene.

### Copy number analysis

TCGA mRNA microarray (Agilent 244 K Custom Gene Expression G4502A-07, Affymetrix Human Genome U133A 2.0 Array, Affymetrix Human Exon 1.0 ST Array) and RNASeqv2 level 3 and clinical data were retrieved from Broad GDAC Firehose http://gdac.broadinstitute.org/. Putative copy-numbers from GISTIC were retrieved from cbio portal (http://www.cbioportal.org/). To find the relationship between FABP4 expression and copy number, we first employed a Shapiro–Wilk test and verified that the data do not follow a normal distribution. The nonparametric Kruskal–Wallis test was applied and no relationship between FABP4 and copy-number could be established. A box-and-whisker plot (Box plot represents first (lower bound) and third (upper bound) quartiles, whiskers represent 1.5 times the interquartile range) was used to visualize the data (log2(x)).

### Correlation of FABP4 with genes from hypoxia metagene signature

The Spearman’s rank-order correlation test was applied to measure the strength of the association between FABP4 and the genes from the Winter et al. hypoxia metagene^20^. We imposed a cut-off of functional relevance on the Spearman correlation coefficient in absolute value of 0.2 based on the method published previously^56^.

### Reverse phase protein analysis

Protein lysates were isolated from HeyA8 MDR cells, 72 h after transfecting them with control siRNA or *FABP4* siRNA. Modified RIPA buffer containing proteinase and phosphatase inhibitor was used for the lysis. Samples were then denatured by 1% SDS with β-mercaptoethanol and were analyzed by MD Anderson Core facility. The heat map was created using normalized log2 median centered values and the data was normalized using the average of the control values. The heat map was generated using http://bioinformatics.mdanderson.org/testchm/.

### Correlation of FABP4 expression with metabolites present in patient tumor samples

The metabolomics data and the gene expression data (normalized log values) were taken from Zand et al., 2016^21–23^. The gene expression was analyzed using qRT-PCR and the metabolites data was analyzed using ultrahigh performance liquid chromatography/tandem mass spectrometry (UHLC/MS/MS2) in those studies. This information was obtained from database. High grade serous ovarian cancer tumor samples (*n* = 61) were analyzed. The samples were divided into high or low FABP4 groups based on the median expression of FABP4 in all samples. Similarly, a median value for each metabolite was calculated for high or low FABP4 samples. The comparison is presented as antilog values.

### DESI-MS imaging

DESI-MS imaging was conducted as described previously^57^. A 2D Omni Spray (Prosolia Inc., Indianapolis, IN) coupled to an LTQ-Orbitrap Elite mass spectrometer (Thermo Scientific, San Jose, CA) was used for tissue imaging. Recently, DESI-MSI has been used to characterize the metabolic profiles of ovarian high-grade serous cancer, borderline serous tumors and normal tissues, and allowed identification of potential predictive markers of ovarian cancer aggressiveness^58^. DESI-MS imaging was performed in the negative and positive ion mode from *m/z* 100–1500, using a hybrid LTQ-Orbitrap mass spectrometer which allows for tandem MS experiments, high mass accuracy (<5 ppm mass error), and high mass resolution (240,000 resolving power) measurements. The spatial resolution of the imaging experiments was 200 µm. Spatially accurate ion images were assembled using BioMap and MSiReader software. The histologically compatible solvent system dimethylformamide:acetonitrile (DMF:ACN) 1:1 (v/v) was used for negative ion mode analysis, at a flow rate of 1.2 µl min^−1^. For positive ion mode analysis, pure ACN was used, at a flow rate of 3 µl min^−1^. The N_2_ pressure was set to 185 psi. For ion identification, high mass resolution/accuracy measurements using the same tissue sections analyzed were conducted. Tandem MS analyses were performed using both the Orbitrap and the linear ion trap for mass analysis.

### Histopathology and light microscopy

The same tissue sections analyzed by DESI-MS imaging were subjected afterwards to standard H&E staining protocol. Light microscopy images of the H&E stained slides were taken using the EVOS FL Auto Cell Imaging System (Invitrogen, Thermo Fisher Scientific, Waltham, MA, USA).

### Survival analysis

Survival analysis was performed in R (version 3.2.5) using Tothill data set. In order to visualize survival (overall or progression free) differences between groups of patients based on FABP4 levels, we used the log-rank test to find the cut-off with the most significant (lowest *p*-value) split into high and low mRNA level groups. Kaplan–Meier plots were generated based on these cutoffs. The median survival time of each group is presented in brackets. The numbers of patients at risk in low and high mRNA groups at different time points are presented at the bottom of the graph. For overall survival, the cut-off to optimally separate the patients in the high versus low (min *p*-value) group was 0.54 for both probes associated to FABP4. For progression free survival, the cut-off to optimally separate the patients in the high versus low (min *p*-value) group was 0.55 for 203980_at and 0.61 for 235978_at.

The relationship between overall survival, respectively progression free survival and covariates (mRNA expression levels and clinical parameters (age and stage)) was examined using a Cox proportional hazard model. A multivariate Cox proportional hazard model was fitted, including the clinical parameters and mRNA expression significant in the univariate analysis.

### Effect of tamoxifen on expression of FABP4

The drug was dissolved in DMSO and physiologically achievable concentrations were used for the experiments. HeyA8 MDR cells were plated in 24 well plates and serum starved before tamoxifen treatment. The cells were collected, and RNA was extracted for qRT-PCR.

### Free fatty acid uptake assay

The experiment was conducted as per the manufacturer’s instructions (Free Fatty Acid Uptake Assay Kit (Fluorometric, ab176768) Cambridge, MA, USA). The cells were serum starved and treated with tamoxifen at a desired concentration. Then the fatty acid dye loading solution was added (100 µl per well of a 96 well plate). The florescence signal was measured at excitation per emission 485 per 515 nm using a bottom read mode after 30 min incubation at room temperature.

### Statistical analysis

MS data corresponding to the areas of interest were extracted using MSiReader software. The *m* per *z* range was discretized by performing hierarchical clustering and cutting the resulting dendrogram at distance 0.05. Peaks appearing in more than 10% of the pixels were kept for analysis. Logistic regression was performed with Lasso regularization using the “glmnet” package (26) in the R language. Regularization parameters were determined by 3-fold cross-validation analysis. The data were randomly equally divided into training and validation sets of samples, 50-50 per patient basis. The training set was used to build a model by cross-validation (CV) based on assigned statistical weights to specific lipid and metabolites important for classification of samples as having either high- or low-FABP4 expression. For other assays, Student’s *t*-test was performed to examine the difference between the control and treatment groups. A *P* value less than 0.05 was deemed statistically significant. All the statistical tests were two-sided. Error bars represent standard error from triplicates.

### Data availability

RPPA data is provided in supplementary data. All other remaining data are available within the article and supplementary files.

## Electronic supplementary material

Supplementary Information

Description of Additional Supplementary Files

Supplementary Data 1
