# Supplementary material for: FABP4 as a key determinant of metastatic potential of ovarian cancer
Source: Nat Commun. 2018 Jul 26;9:2923. doi: 10.1038/s41467-018-04987-y (PMC6062524; doi:10.1038/s41467-018-04987-y)
Supplement: Supplementary file 1 — Supplementary Information [file 41467_2018_4987_MOESM1_ESM.docx]

File Name: Supplementary Information
Description: Supplementary Figures and Supplementary Tables


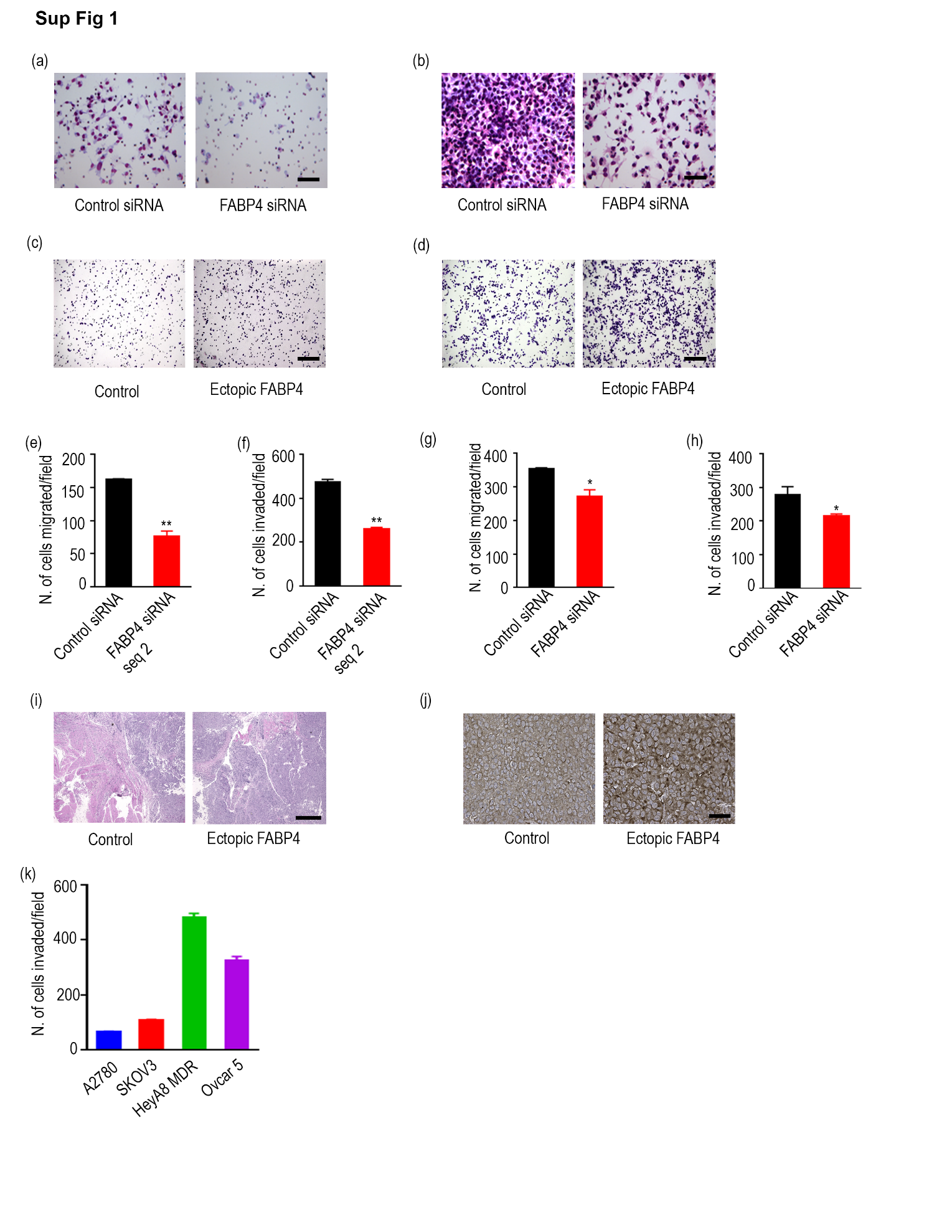


**Supplementary Figure 1. Effects of FABP4 on ovarian tumor progression *in vitro* and *in vivo*.** (a,b) Representative images of (a) migration and (b) invasion assay results in HeyA8 MDR cells transfected with control or FABP4 siRNA. (c,d) Representative images of (c) migration and (d) invasion assay results in A2780-ip1 cells transfected with control or FABP4 vector. (e,f) Effect of FABP4 knockdown on (e) migration and (f) invasiveness using a second siRNA sequence. **p<0.01. n=3 (g,h) Effect of FABP4 knockdown on (g) migration and (h) invasion in Ovcar 5 cells. *p < 0.05. n=3 (i) Representative hematoxylin and eosin-stained sections of tumor tissues from mice injected with control A2780-ip1 cells or A2780-ip1 cells transfected with FABP4-expressing vector. (j) Immunohistochemical expression of FABP4 in tumor tissues from mice injected with control A2780-ip1 cells or A2780-ip1 cells with ectopic expression of FABP4. (k) Basal invasion potential of A2780, SKOV3ip1 (Cell lines with low expression of FABP4), HeyA8MDR and Ovcar 5 cell lines (Cell lines with high expression of FABP4).


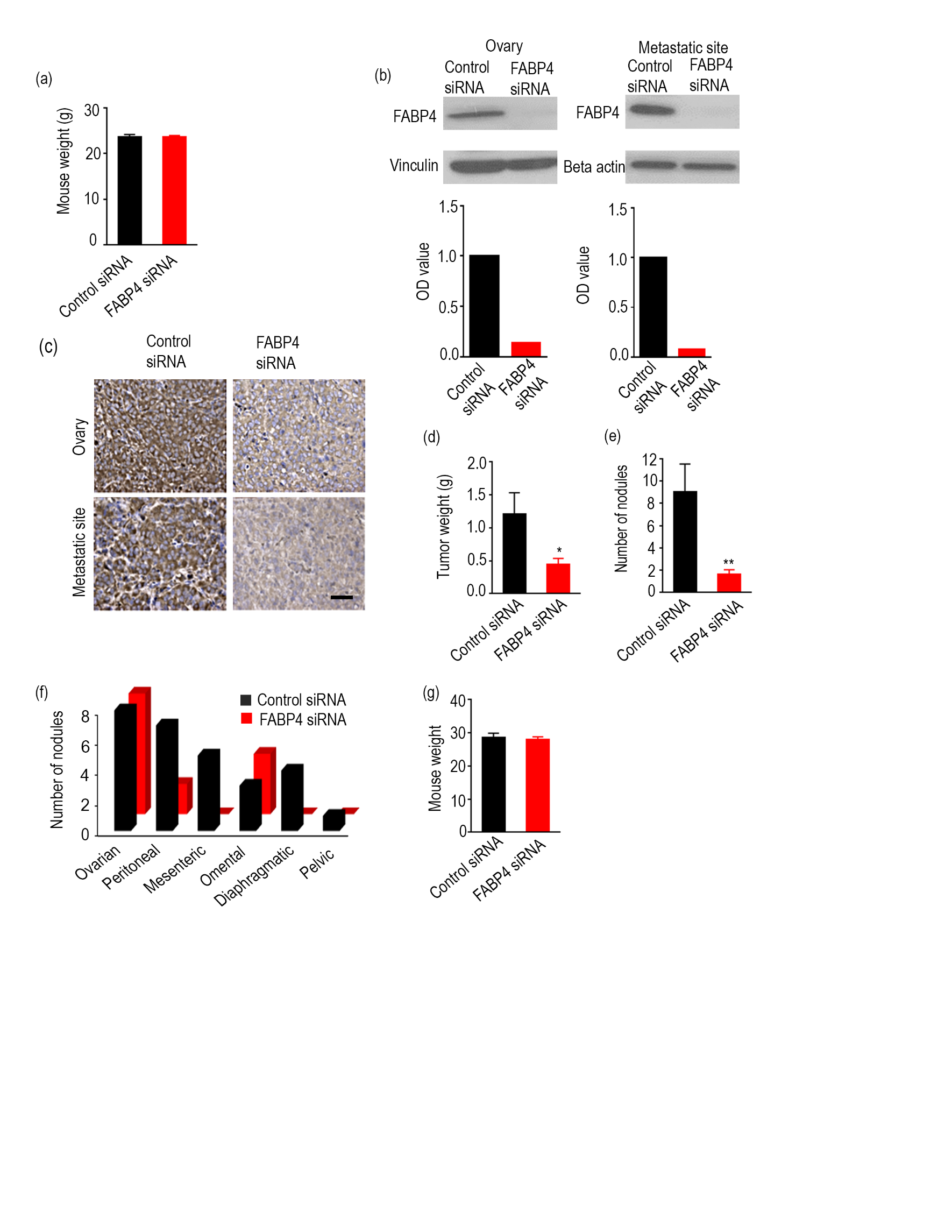


**Supplementary Figure 2. Effect of knockdown of FABP4 on tumor progression.**  (a) Mouse weight in the control siRNA and FABP4 siRNA groups. (b) FABP4 protein expression levels at the primary site (ovary) and metastatic sites after treatment with control or FABP4 siRNA, as shown in Western blot analysis. OD-Optical Density (c) Immunohistochemical staining showing FABP4 protein expression levels at the primary site (ovary) and metastatic sites after treatment with control or FABP4 siRNA. (d) Aggregate tumor weight for mice injected with Ovcar 5 cell line. The mice were treated with control siRNA or FABP4 siRNA encapsulated in DOPC liposomes. *p<0.05. (e) Effect of knockdown of FABP4 on the average number of nodules. **p<0.01. (f) Pattern of metastasis in the control and treated mice. (g) Mouse weight in the control and treatment groups.


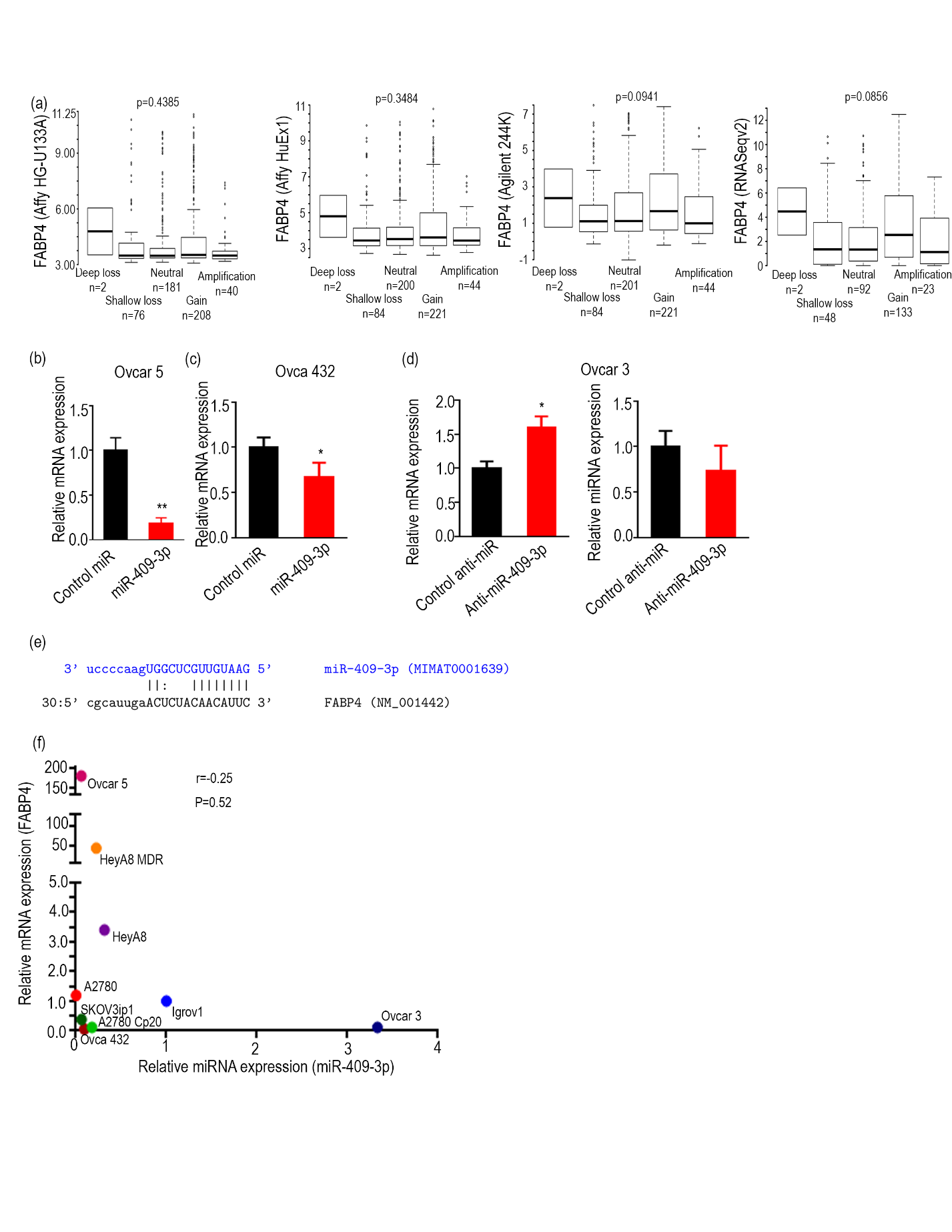


**Supplementary Figure 3. Upstream regulation of FABP4.** (a) Correlation between copy number and mRNA expression levels of FABP4 across platforms (Agilent and Affymetrics, RNAseqv2) using data from The Cancer Genome Atlas. (b) Effect of miR-409-3p mimic transfection on the expression of FABP4 in Ovcar 5 cells. **p<0.01. (c) Effect of miR-409-3p mimic transfection on the expression of FABP4 in Ovca 432 cells. *p<0.05. (d) Effect of miR-409-3p inhibitor on the expression of FABP4 mRNA (left) and on the level of miR-409-3p (right) in Ovcar 3 cells. *p < 0.05. (e) Predicted binding site of miR-409-3p on the 3’ untranslated region (UTR) of FABP4. (f) Inverse association between FABP4 and miR-409-3p in ovarian cancer cell lines.


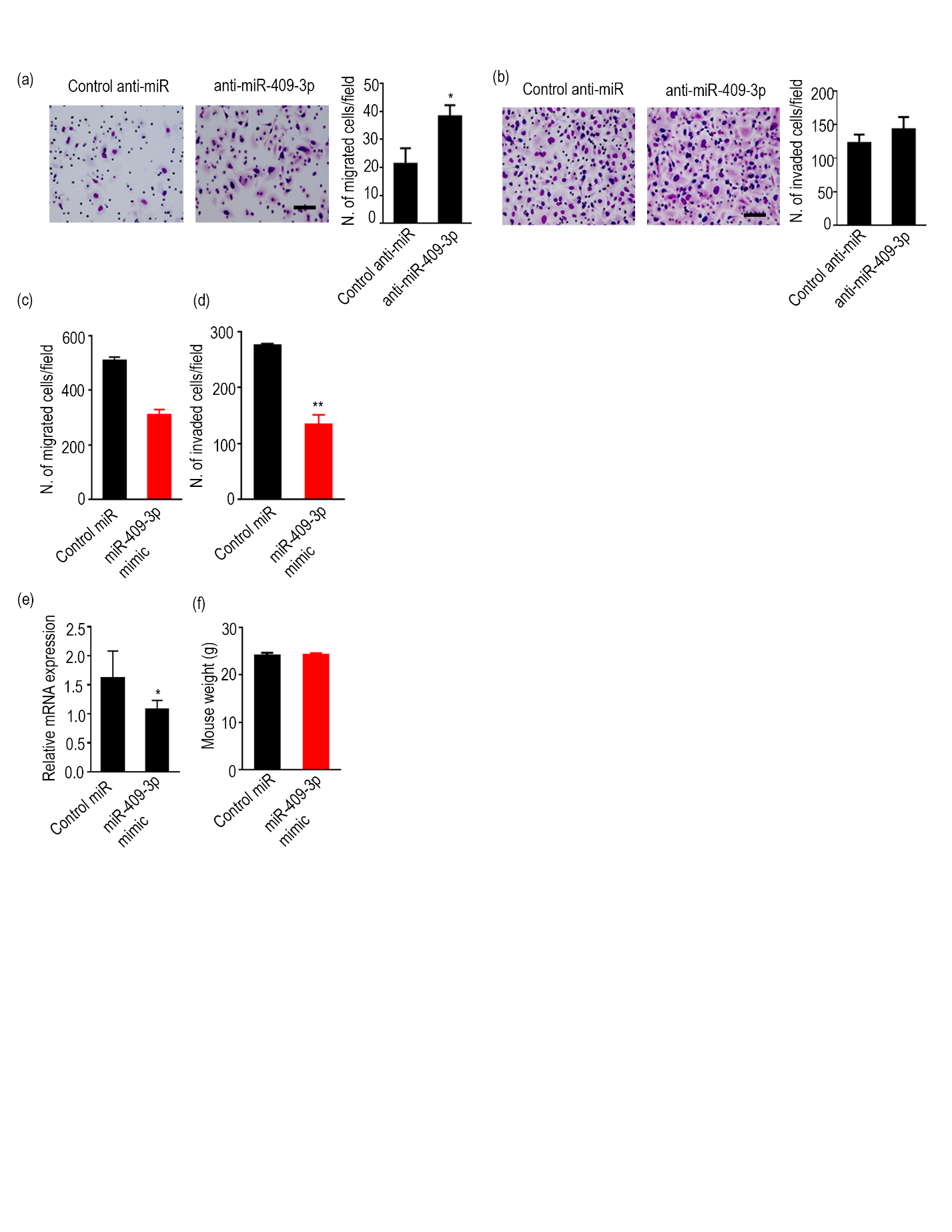


**Supplementary Figure 4. Effect of miR-409-3p on tumor progression and FABP4.** (a,b) Effect of anti-miR-409-3p transfection on (a) migration and (b) invasiveness of Ovcar 3 cells. *p < 0.05. (c, d) Effect of miR-409-3p mimic transfection on (c) migration and (d) invasiveness of Ovcar 5 cells. **p < 0.01. (e) Expression of FABP4 in tumor tissues after treatment with miR-409-3p mimic treatment. *p < 0.05. (f) The weight of the control and treated mice at the end of the experiment.

**
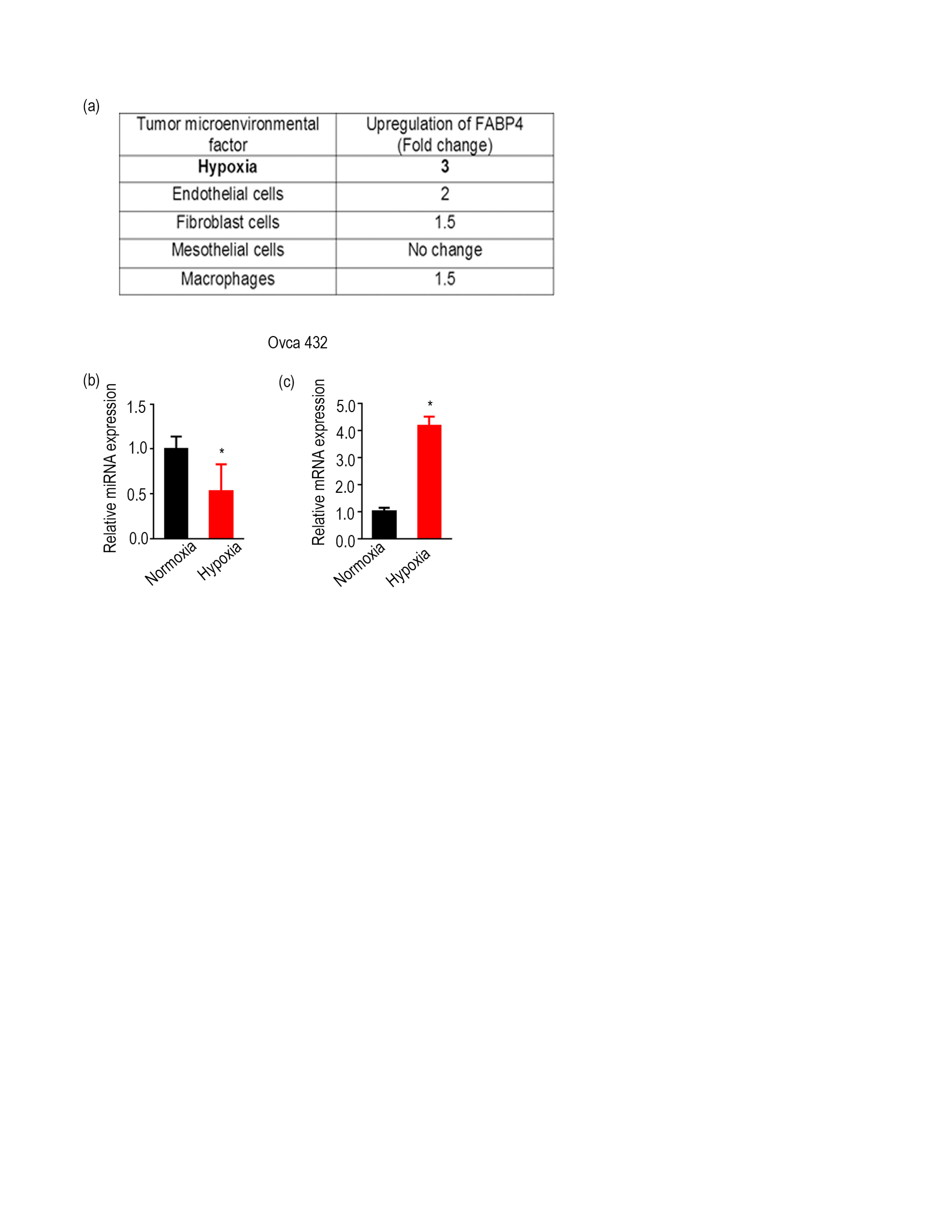
Supplementary Figure 5. Tumor microenvironmental factors in the regulation of miR-409-3p and FABP4.** (a) Effect of tumor microenvironmental factors on the expression of FABP4. (b, c) Effect of hypoxia on (b) miR-409-3p expression and (c) FABP4 expression levels in Ovca 432 cells. *p < 0.05.


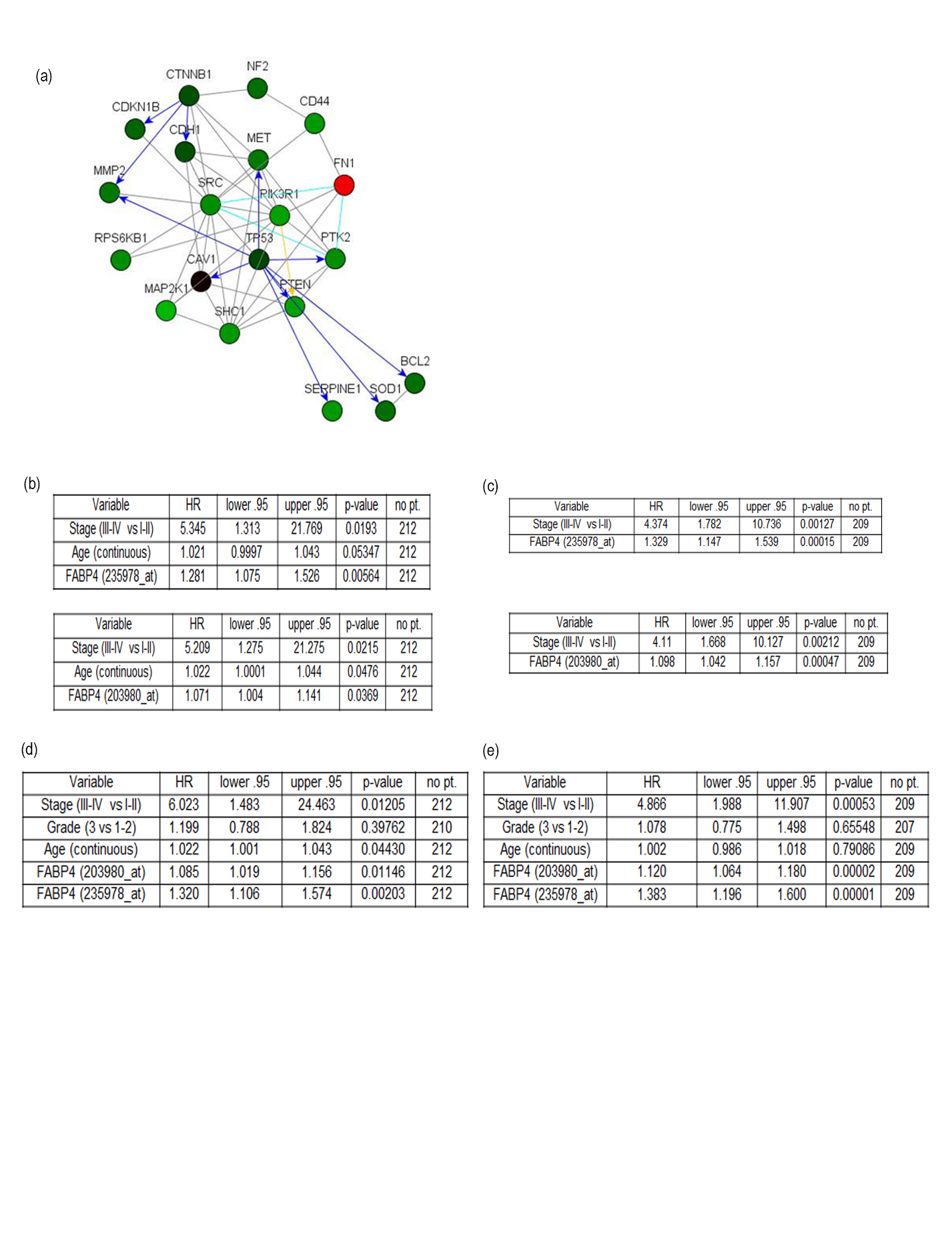


**Supplementary Figure 6. Downstream effect of FABP4** (a) Proteins that were downregulated in the *FABP4* siRNA group are related to the metastasis pathway. (b, c) Multivariate analysis for (b) overall and (c) progression-free survival (d, e) Univariate analysis for (d) overall and (e) progression-free survival based on FABP4 expression in ovarian cancer patients. The Data were extracted from Tothill data set.

**
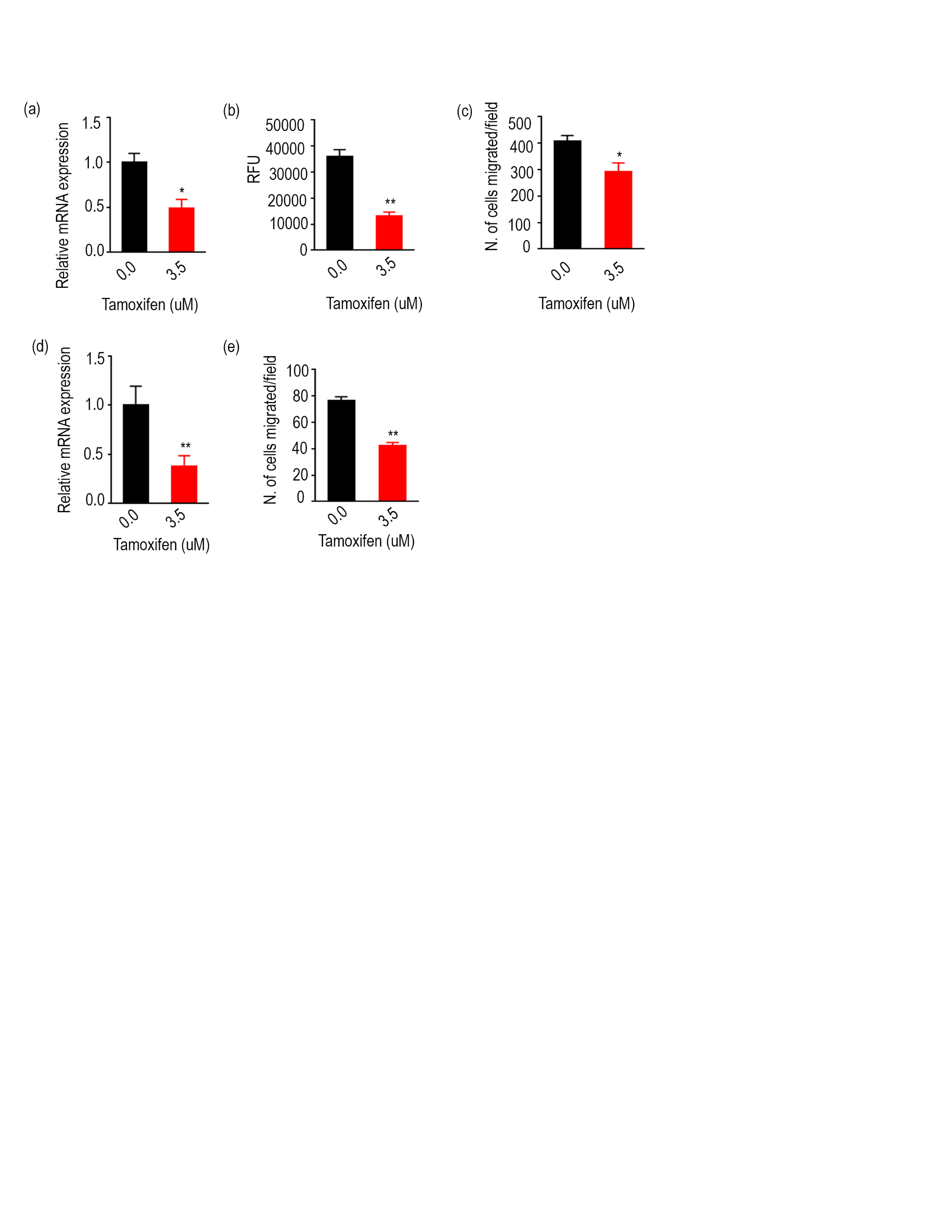
**

**Supplementary Figure 7.** **Clinically approved drugs for FABP4 inhibition and additional cancer model.** (a) Effect of tamoxifen treatment on the expression of FABP4 in HeyA8 MDR cells. *p<0.05. (b) Effect of tamoxifen treatment on the ability of cancer cells to take up free fatty acids (HeyA8 MDR cells). **p<0.01. (c) Effect on the migratory potential of cancer cells after tamoxifen treatment (HeyA8 MDR cells). *p<0.05. (d) Effect of tamoxifen treatment on the expression of FABP4 in A2780 cells. **p<0.01. (e) Effect on migration of A2780 cells after tamoxifen treatment. N=3 (**p<0.01).


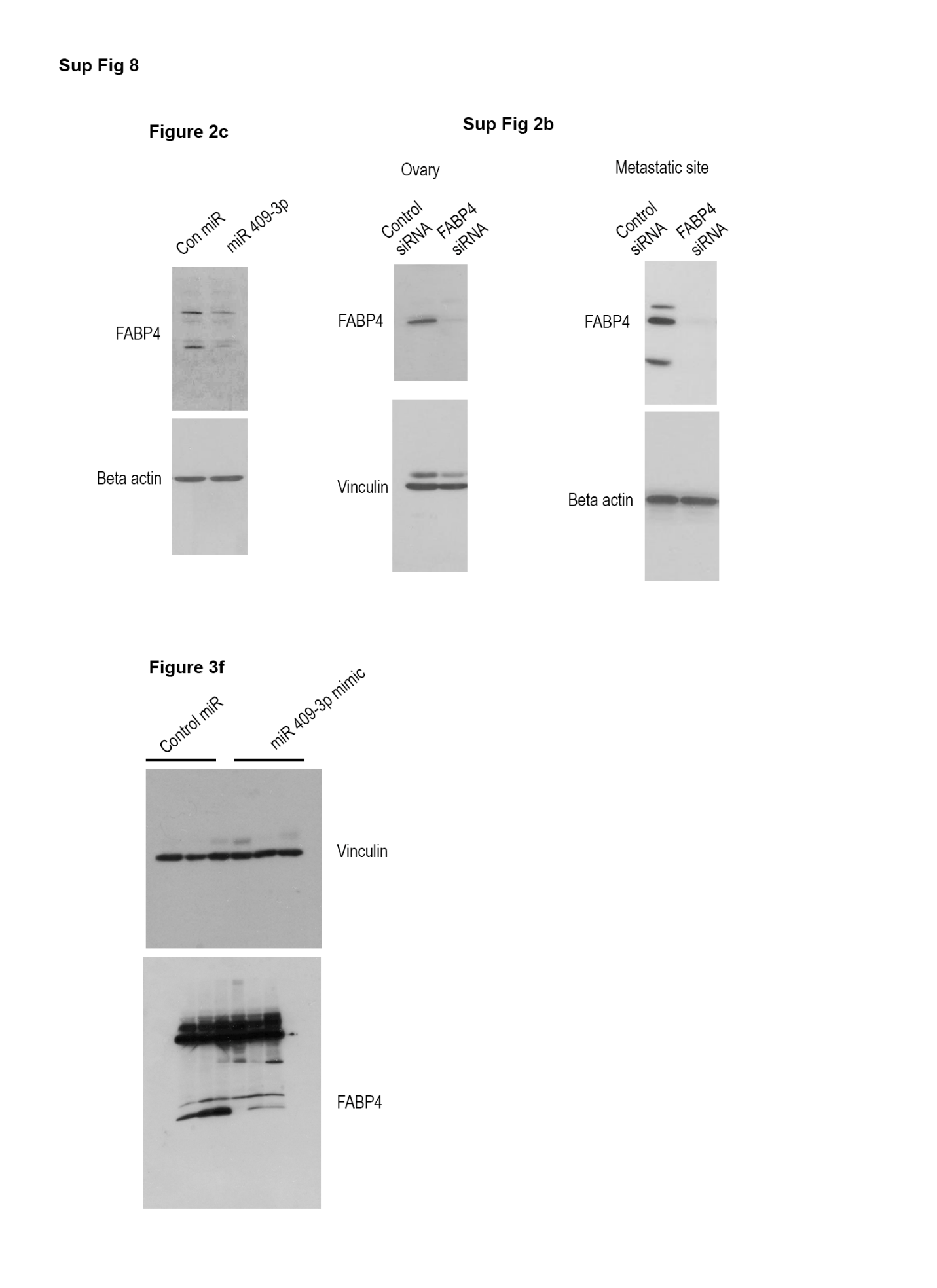


**Supplementary Figure 8.** Uncropped scans of blots.

| **MicroRNA** | **MIC score** |
| --- | --- |
| hsa-miR-143 | 0.270601 |
| hsa-miR-126 | 0.22939 |
| hsa-miR-214* | 0.22323 |
| hsa-let-7g* | 0.22284 |
| hsa-miR-199a-5p | 0.22044 |
| **hsa-miR-409-3p** | **0.21971** |
| hsa-miR-507 | 0.21927 |
| hsa-miR-145* | 0.21636 |
| hsa-miR-214 | 0.21551 |
| hsa-miR-1225-3p | 0.21542 |
| hsa-miR-431 | 0.21531 |
| hsa-miR-342-3p | 0.21100 |
| hsa-miR-199b-5p | 0.21077 |
| hsa-miR-145 | 0.21002 |
| hsa-miR-150 | 0.20990 |
| hsa-miR-133a | 0.20808 |
| hsa-miR-409-5p | 0.20784 |
| hsa-miR-199b-3p | 0.20771 |
| hsa-miR-22 | 0.20767 |
| hsa-miR-22* | 0.20705 |
| hsa-miR-133b | 0.20703 |
| hsa-miR-1 | 0.20599 |
| hsa-miR-1225-5p | 0.20435 |
| hsa-miR-514 | 0.20427 |
| hsa-miR-132 | 0.20395 |
| hsa-miR-152 | 0.20356 |
| hsa-miR-432 | 0.20268 |
| hsa-miR-508-3p | 0.20240 |
| hsa-miR-139-5p | 0.20203 |
| hsa-miR-379 | 0.20181 |
| hsa-miR-182 | 0.20142 |
| hsa-miR-143* | 0.20019 |

**Supplementary Table 1.** miRNAs with MIC scores greater than 0.2 for association with FABP4 gene expression

**
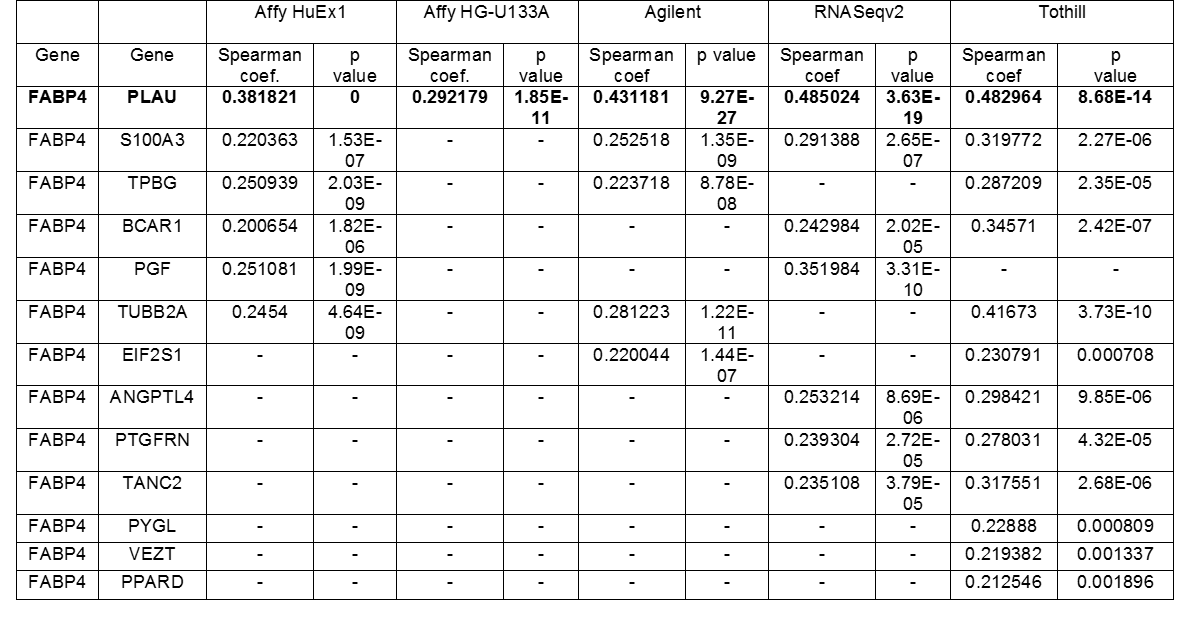
**

**Supplementary Table 2.** Correlation of FABP4 gene expression with the expression of genes listed in Winter hypoxia metagene signature across various data platforms

| **BIOCHEMICAL NAME** | **Antilog** | **BIOCHEMICAL NAME** | **Antilog** |
| --- | --- | --- | --- |
| glutathione, reduced (GSH) | 0.019 | docosatrienoate-22-3n3- | 0.954 |
| N-acetylaspartate (NAA) | 0.154 | N-acetylglycine | 0.955 |
| glycerophosphorylcholine (GPC) | 0.275 | proline | 0.955 |
| glycerol 3-phosphate (G3P) | 0.301 | phenylalanine | 0.956 |
| ascorbate (Vitamin C) | 0.319 | histidine | 0.956 |
| N-acetyl-aspartyl-glutamate-NAAG- | 0.494 | aspartate | 0.958 |
| glucose 6-phosphate (G6P) | 0.511 | pipecolate | 0.959 |
| glutathione, oxidized (GSSG) | 0.543 | N-acetylneuraminate | 0.959 |
| fructose-6-phosphate | 0.563 | serine | 0.960 |
| 1-palmitoylglycerophosphoethanolamine | 0.566 | 1,5-anhydroglucitol (1,5-AG) | 0.961 |
| adenosine-3-monophosphate-3 (AMP) | 0.571 | valine | 0.963 |
| dehydroascorbate | 0.579 | glycolate (hydroxyacetate) | 0.964 |
| Isobar-ribulose-5-phosphate-xylulose-5-phosphate | 0.581 | C-glycosyltryptophan- | 0.967 |
| 1-arachidonoylglycerophosphoinositol- | 0.587 | mannose | 0.969 |
| 2-oleoylglycerophosphoethanolamine- | 0.596 | mannitol | 0.973 |
| glycerol 2-phosphate | 0.602 | 3-dehydrocarnitine* | 0.973 |
| 1-palmitoylplasmenylethanolamine- | 0.614 | 2-aminoadipate | 0.976 |
| 1-oleoylglycerophosphoethanolamine | 0.614 | gamma-glutamylphenylalanine | 0.976 |
| dihydroxyacetone-phosphate-DHAP- | 0.616 | stearate (18:0) | 0.977 |
| S-adenosylhomocysteine (SAH) | 0.622 | ribulose | 0.978 |
| p-cresol-sulfate | 0.677 | 5,6-dihydrouracil | 0.981 |
| phosphoenolpyruvate (PEP) | 0.678 | pelargonate (9:0) | 0.981 |
| mannose-6-phosphate | 0.682 | gamma-glutamylglutamine | 0.982 |
| 1-palmitoylglycerophosphoinositol- | 0.687 | tyrosine | 0.985 |
| 2-hydroxystearate | 0.703 | putrescine | 0.985 |
| carnitine | 0.707 | lactate | 0.985 |
| gluconate | 0.736 | uracil | 0.988 |
| 2-hydroxypalmitate | 0.737 | phosphoethanolamine | 0.988 |
| choline-phosphate | 0.740 | erythritol | 0.991 |
| 1-stearoylglycerophosphoethanolamine | 0.740 | urate | 0.991 |
| butyrylcarnitine | 0.740 | hexaethylene-glycol | 0.992 |
| docosahexaenoate (DHA; 22:6n3) | 0.741 | N-acetylserine | 0.992 |
| behenate-22-0- | 0.748 | ornithine | 0.994 |
| 2-phosphoglycerate | 0.752 | phosphate | 0.995 |
| 7-alpha-hydroxy-3-oxo-4-cholestenoate-7-Hoca- | 0.755 | sorbitol | 0.996 |
| 4-hydroxybutyrate-GHB- | 0.757 | ribose-5-phosphate | 0.996 |
| 2-hydroxyglutarate | 0.764 | cystine | 0.997 |
| xylulose | 0.779 | caprylate (8:0) | 0.998 |
| erythronate* | 0.781 | margarate (17:0) | 0.999 |
| 1-linoleoylglycerophosphoethanolamine- | 0.785 | 3-carboxy-4-methyl-5-propyl-2-furanpropanoate (CMPF) | 1.000 |
| propionylcarnitine | 0.786 | biliverdin | 1.000 |
| ribitol | 0.786 | tetradecanedioate | 1.000 |
| 1-stearoylglycerophosphoinositol | 0.787 | ethanolamine | 1.001 |
| inosine | 0.787 | asparagine | 1.001 |
| 6-phosphogluconate | 0.789 | myristate (14:0) | 1.010 |
| dihomo-linolenate-20-3n3-or-n6- | 0.791 | hydroxyisovaleroyl-carnitine | 1.011 |
| adrenate (22:4n6) | 0.793 | N-acetylthreonine | 1.011 |
| arachidonate (20:4n6) | 0.794 | palmitoyl-ethanolamide | 1.015 |
| docosapentaenoate-n6-DPA-22-5n6- | 0.795 | taurine | 1.016 |
| ribose | 0.796 | gamma-aminobutyrate (GABA) | 1.017 |
| 1,3-dihydroxyacetone | 0.797 | arginine | 1.018 |
| phenylacetylglutamine | 0.817 | N6-acetyllysine | 1.019 |
| 3-phosphoglycerate | 0.820 | laurate (12:0) | 1.019 |
| spermidine | 0.821 | creatinine | 1.022 |
| alpha-hydroxyisovalerate | 0.823 | N-acetylalanine | 1.023 |
| bilirubin*E-E- | 0.825 | hypotaurine | 1.023 |
| adenosine-2-monophosphate-2 (AMP) | 0.830 | assymetric-dimethylarginine (ADMA) | 1.023 |
| isobutyrylcarnitine | 0.833 | nonadecanoate (19:0) | 1.027 |
| gamma-glutamylglutamate | 0.837 | methylphosphate | 1.027 |
| 1-oleoylglycerophosphoinositol- | 0.838 | tryptophan | 1.027 |
| isovalerylcarnitine | 0.839 | docosadienoate-22-2n6- | 1.030 |
| pyruvate | 0.840 | myristoleate (14:1n5) | 1.030 |
| palmitoyl-sphingomyelin | 0.843 | lysine | 1.034 |
| ophthalmate | 0.844 | argininosuccinate | 1.038 |
| arabitol | 0.846 | glycerol | 1.039 |
| 4-androsten-3beta-17beta-diol-disulfate-1- | 0.852 | cysteine | 1.039 |
| guanosine | 0.854 | ergothioneine | 1.041 |
| malate | 0.859 | caprate (10:0) | 1.045 |
| 3-(4-hydroxyphenyl)lactate | 0.862 | trans-4-hydroxyproline | 1.048 |
| succinylcarnitine | 0.863 | scyllo-inositol | 1.049 |
| 3-aminoisobutyrate | 0.864 | eicosenoate (20:1n9 or 11) | 1.050 |
| fumarate | 0.864 | riboflavin (Vitamin B2) | 1.057 |
| flavin adenine dinucleotide (FAD+) | 0.866 | gamma-glutamylvaline | 1.060 |
| 1-arachidonoylglycerophosphoethanolamine- | 0.867 | fructose-1-phosphate | 1.062 |
| 2-hydroxybutyrate (AHB) | 0.871 | 3-hydroxybutyrate (BHBA) | 1.064 |
| pantothenate | 0.872 | palmitate (16:0) | 1.064 |
| eicosapentaenoate (EPA; 20:5n3) | 0.874 | docosapentaenoate (n3-DPA; 22:5n3) | 1.068 |
| beta-hydroxyisovalerate | 0.876 | maltose | 1.070 |
| caproate (6:0) | 0.878 | dihomo-linoleate-20-2n6- | 1.074 |
| glycylglycine | 0.880 | N-acetylmethionine | 1.076 |
| cholesterol | 0.880 | N-acetylornithine | 1.086 |
| undecanedioate | 0.881 | 15-HETE | 1.100 |
| glycerate | 0.882 | citrate | 1.111 |
| N1-methyladenosine | 0.887 | pseudouridine | 1.116 |
| 2-aminobutyrate | 0.887 | stearidonate (18:4n3) | 1.118 |
| inositol-1-phosphate (I1P) | 0.888 | 15-methylpalmitate-isobar-with-2-methylpalmitate- | 1.119 |
| uridine | 0.889 | pentadecanoate (15:0) | 1.123 |
| pyroglutamine* | 0.889 | beta-alanine | 1.125 |
| pregnen-diol-disulfate- | 0.891 | 13-HODE-9-HODE | 1.126 |
| nicotinamide | 0.896 | cortisol | 1.129 |
| choline | 0.896 | 3-methyl-2-oxovalerate | 1.129 |
| glutamine | 0.898 | succinate | 1.133 |
| alanine | 0.900 | oleate (18:1n9) | 1.138 |
| cytidine 5'-monophosphate-5-CMP- | 0.902 | 4-methyl-2-oxopentanoate | 1.153 |
| cysteine-glutathione-disulfide | 0.904 | 2-linoleoylglycerophosphoethanolamine- | 1.160 |
| threonine | 0.906 | N-acetylglucosamine-6-phosphate | 1.165 |
| hypoxanthine | 0.907 | betaine | 1.171 |
| xanthosine | 0.907 | glucose | 1.173 |
| xanthine | 0.908 | alpha-tocopherol | 1.174 |
| acetylphosphate | 0.919 | octadecanedioate | 1.185 |
| pyrosphosphate (PPi) | 0.923 | linoleate (18:2n6) | 1.193 |
| leucine | 0.926 | Isobar-fructose-1,6-diphosphate-glucose-1,6-diphosphate-myo-inositol-1-4-or-1,3-diphosphate | 1.199 |
| isoleucine | 0.926 | phenol sulfate | 1.209 |
| cystathionine | 0.927 | 10-nonadecenoate (19:1n9) | 1.225 |
| myo-inositol | 0.928 | guanine | 1.236 |
| kynurenine | 0.934 | hexadecanedioate | 1.239 |
| hippurate | 0.937 | linolenate [alpha or gamma] (18:3n3 or 6) | 1.257 |
| urea | 0.938 | palmitoleate (16:1n7) | 1.262 |
| heptaethylene-glycol | 0.938 | 1-2-propanediol | 1.270 |
| methionine | 0.939 | threonate | 1.273 |
| gamma-glutamylleucine | 0.940 | maltotriose | 1.302 |
| acetylcarnitine | 0.940 | 17-methylstearate | 1.355 |
| creatine | 0.942 | stachydrine | 1.382 |
| fructose | 0.943 | 10-heptaadecenoate (17:1n7) | 1.389 |
| glutamate | 0.945 | 1-stearoylglycerophosphocholine | 1.436 |
| N-formylmethionine | 0.946 | maltotetraose | 1.637 |
| tryptophan-betaine- | 0.948 | 2-docosahexaenoylglycerophosphoethanolamine- | 1.673 |
| 5-oxoproline | 0.951 | 2-arachidonoylglycerophosphocholine* | 1.751 |
| glycine | 0.953 | 1-palmitoylglycerophosphocholine | 2.544 |
| azelate (nonanedioate) | 0.954 | 2-arachidonoylglycerophosphoethanolamine- | 6.094 |

**Supplementary Table 3.** Metabolite species expressed in high or low FABP4 expressing patient tumor samples.

| **LOW FABP4 EXPRESSION** | | **HIGH FABP4 EXPRESSION** | |
| --- | --- | --- | --- |
| **Attribution** | **SAM Score** | **Attribution** | **SAM Score** |
| ***Metabolites*** |  | ***Metabolites*** |  |
| **Fumarate** | **18.1** | **N-acetylaspartic acid** | **-17.2** |
| **Acetyl-glycine** | **11.1** | **Ascorbic acid** | **-5.9** |
| **Succinate** | **31.0** | **Gluconic acid** | **-41.9** |
| **2-Hydroxy-3-methylbutyric acid** | **19.1** | **Phosphatidic acid** | **-6.8** |
| **Taurine** | **27.3** | ***Fatty Acids*** |  |
| **Pyroglutamate** | **8.3** | **FA 14:1** | **-23.7** |
| **Aspartate** | **18.9** | **FA 17:0** | **-0.5** |
| **3-Hydroxypicolinic acid** | **40.8** | **FA hydroxy 16:0** | **-13.8** |
| **Glutamine** | **20.8** | **FA 18:3** | **-4.1** |
| **Glutamic acid** | **26.9** | **FA 18:2** | **-11.1** |
| **2-Hydroxyglutarate** | **21.0** | **FA 18:1** | **-5.4** |
| **Xanthine** | **37.7** | **FA 18:0** | **-3.6** |
| **Glycerophosphoglycerol** | **18.4** | **FA 20:5** | **-12.2** |
| **Glutathione** | **42.8** | **FA 20:4** | **-27.4** |
| ***Fatty Acids*** |  | **FA 18:2** | **-17.2** |
| **FA 8:0** | **29.7** | **FA 18:1** | **-21.3** |
| **FA 9:0** | **29.8** | **FA hydroxy 20:4** | **-18.9** |
| **FA 15:0** | **4.9** | **FA 18:0** | **-22.2** |
| **FA 16:1** | **3.5** | **FA hydroxy 20:3** | **-8.6** |
| **FA 20:3** | **15.1** | **FA 22:6** | **-21.3** |
| **FA 20:2** | **11.2** | **FA 22:5** | **-22.6** |
| **FA 20:1** | **20.2** | **FA 22:4** | **-37.6** |
| **FA 20:0** | **8.8** | **FA 22:3** | **-6.2** |
| **FA 22:0** | **10.8** | **FA 20:4** | **-32.2** |
| **FA 23:1** | **11.2** | **FA hydroxy 22:6** | **-6.0** |
| **FA 23:0** | **7.6** | **FA 24:5** | **-16.6** |
| **FA 24:1** | **21.3** | **FA 24:4** | **-17.5** |
| **FA 24:0** | **21.7** | **FA 24:3** | **-0.9** |
| **FA 26:3** | **11.4** | **FA 22:4** | **-33.4** |
| **FA 26:2** | **17.7** | **FA hydroxy 24:0** | **-2.8** |
| **FA 26:1** | **11.7** | ***Glycerolipids*** |  |
| **FA 26:0** | **29.9** | **MG 18:0** | **-7.5** |
| ***Glycerolipids*** |  | **MG 22:6** | **-6.8** |
| **DG 24:0/0:0** | **10.5** | **DG 32:1/0:0** | **-8.2** |
| ***Glycerophosphoethanolamines*** |  | **DG 32:0/0:0** | **-3.5** |
| **PE 18:1/1:0** | **11.0** | **DG 34:3/0:0** | **-2.3** |
| **PE 20:3/1:0** | **21.3** | **DG 34:2/0:0** | **-6.7** |
| **PE O-34:3 or PE P-34:2** | **23.0** | **DG 34:1/0:0** | **-14.2** |
| **PE O-34:2 or PE P-34:1** | **21.5** | **DG 36:4/0:0** | **-13.3** |
| **PE 34:2** | **25.3** | **DG 36:3/0:0** | **-4.8** |
| **PE 34:1** | **21.4** | **DG 36:2/0:0** | **-15.7** |
| **PE O-36:3 or P-36:2** | **15.8** | **DG 38:6/0:0** | **-18.9** |
| **PE 35:1** | **9.6** | **DG 38:5/0:0** | **-16.0** |
| **PE 36:3** | **27.4** | **DG 38:4/0:0** | **-15.4** |
| **PE 36:2** | **18.3** | **DG 40:7/0:0** | **-2.8** |
| **PE 38:3** | **27.7** | **DG 40:6/0:0** | **-15.6** |
| ***Glycerophosphoglycerols*** |  | **DG 40:5/0:0** | **-15.1** |
| **PG 18:0/18:1** | **19.9** | ***Glycerophosphoethanolamines*** |  |
| **PG 18:0/18:0** | **13.6** | **LysoPE P-16:0** | **-3.5** |
| **PG 38:4** | **6.5** | **LysoPE O-18:1** | **-30.4** |
| **PG 38:3** | **14.0** | **LysoPE 18:1** | **-4.2** |
| **PG 38:2** | **12.2** | **LysoPE 18:0** | **-5.8** |
| **PG 42:7** | **9.5** | **LysoPE 20:4** | **-18.6** |
| ***Ceramides*** |  | **PE 20:4/1:0** | **-36.2** |
| **Cer d18:16:0** | **13.1** | **PE 34:0** | **-0.7** |
| **Cer m18:1/22:0** | **7.3** | **PE P-18:0/18:4** | **-10.5** |
| **Cer m18:1/24:1** | **20.0** | **PE O-38:5 or PE P-38:4** | **-35.7** |
| **Cer m42:1** | **16.9** | **PE 38:5** | **-24.4** |
| **Cer d18:1/23:0** | **5.7** | **PE 38:4** | **-6.4** |
| **Cer d40:0(2OH)** | **16.9** | **PE 38:2** | **-4.1** |
| **Cer d42:1** | **10.0** | **PE 38:1** | **-19.2** |
| **Cer d42:0** | **21.9** | **PE 39:6** | **-17.6** |
| **Cer d18:1/25:0** | **11.7** | **PE 39:5** | **-17.6** |
| **Cer d18:1/26:1** | **11.4** | **PE O-40:5 or PE P-40:4** | **-20.1** |
| **Cer d18:1/26:0** | **6.8** | **PE 40:5** | **-10.2** |
| **PE - Cer 36:3** | **11.3** | **PE 37:1** | **-4.9** |
| **GlcCer d34:1** | **5.9** | **PE 40:4** | **-20.8** |
| **PE-Cer d37:1** | **14.7** | **PE 39:4** | **-4.4** |
| ***Monoacylglycerophosphates*** |  | **PE 39:2** | **-12.7** |
| **PA 32:0** | **17.3** | **PE 39:1** | **-16.6** |
| **PA 24:2** | **17.9** | **PE 41:6** | **-14.7** |
| **PA 24:1** | **22.3** | **PE 41:5** | **-26.4** |
| **PA 36:4** | **11.9** | **PE 41:4** | **-25.0** |
| **PA 36:3** | **12.8** | **PE 43:6** | **-7.3** |
| ***Cardiolipins*** |  | **PE 43:2** | **-8.2** |
| **CL 68:5** | **5.2** | ***Glycerophosphoglycerols*** |  |
| **CL 70:7** | **25.3** | **LysoPG 16:0** | **-7.1** |
| **CL 70:6** | **20.9** | **LysoPG 18:2** | **-20.2** |
| **CL 70:5** | **11.3** | **LysoPG 18:1** | **-18.4** |
| **CL 72:8** | **20.7** | **LysoPG 22:6** | **-11.5** |
| **CL 72:7** | **26.9** | **PG 34:3** | **-0.7** |
| **CL 72:6** | **26.0** | **PG 34:2** | **-1.2** |
| **CL 74:10** | **13.3** | **PG 16:0/18:1** | **-4.3** |
| **CL 74:9** | **22.1** | **PG 36:4** | **-18.5** |
| **CL 74:8** | **22.5** | **PG 18:2/18:1** | **-4.8** |
| **CL 74:7** | **18.9** | **PG 18:1/18:1** | **-2.3** |
| **CL 76:10** | **19.9** | **PG 38:6** | **-7.6** |
| **CL 76:9** | **15.0** | **PG 38:5** | **-2.8** |
| **CL 76:8** | **14.3** | **PG 40:8** | **-6.2** |
| **CL 76:7** | **13.4** | **PG 40:7** | **-2.8** |
| **CL 36:4** | **2.8** | **PG 40:6** | **-0.7** |
| ***Glycerophosphoinositols*** |  | **PG 40:5** | **-0.7** |
| **LysoPI (16:0/0:0)** | **4.4** | **PG 22:6/22:6** | **-8.0** |
| **PI (32:1)** | **22.3** | ***Ceramides*** |  |
| **PI (32:0)** | **25.4** | **Cer d32:1** | **-8.9** |
| **PI (34:2)** | **28.1** | **Cer d16:1/17:0** | **-16.2** |
| **PI (34:1)** | **25.5** | **Cer d34:2** | **-8.5** |
| **PI (O-23:0)** | **7.8** | **Cer d34:1** | **-1.1** |
| **PI (25:1)** | **21.5** | **Cer d36:2** | **-4.7** |
| **PI (36:4)** | **2.9** | **Cer d36:1** | **-17.6** |
| **PI (36:3)** | **31.7** | **Cer d38:1** | **-8.2** |
| **PI (36:2)** | **45.5** | **Cer d41:2** | **-1.3** |
| **PI (36:1)** | **25.7** | **Cer d42:0** | **-0.5** |
| **PI (37:3)** | **17.9** | **Cer d42:1** | **-1.2** |
| **PI (38:6)** | **20.6** | **PE-Cer d36:1** | **-4.1** |
| **PI (38:3)** | **18.7** | ***Monoacylglycerophosphates*** | |
| **PI (40:6)** | **9.9** | **PA 36:1** | **-18.2** |
| **PI (39:4)** | **20.0** | **PA O-38:2 or PA P-38:1** | **-11.9** |
| ***Glycerophosphoserines*** |  | ***Cardiolipins*** |  |
| **PS P-34:1** | **4.3** | **CL 72:4** | **-7.5** |
| **PS 16:0/18:1** | **18.6** | ***Glycerophosphoinositols*** |  |
| **PS P-36:2 or PS O-36:3** | **14.4** | **LysoPI 15:0** | **-16.3** |
| **PS O-36:2 or PS P-36:1** | **4.3** | **LysoPI O-16:0** | **-5.8** |
| **PS 36:3** | **28.2** | **LysoPI 18:0** | **-20.1** |
| **PS 36:2** | **34.3** | **Lyso PI 20:4** | **-15.7** |
| **PS 18:0/18:1** | **17.5** | **PI O-33:2 or PI P-33:1** | **-13.0** |
| **PS O-38:4 or P-38:3** | **4.3** | **PI P-18:0/17:2** | **-15.2** |
| **PS 38:3** | **31.9** | **PI O-35:2 or PI P-35:1** | **-1.0** |
| **PS 38:2** | **30.3** | **PI 37:4** | **-3.6** |
| **PS 38:1** | **23.5** | **PI 38:6** | **-15.6** |
| **PS 40:6** | **13.3** | **PI 38:5** | **-15.2** |
| **PS 40:2** | **17.7** | **PI 38:4** | **-27.6** |
| **PS 40:1** | **21.3** | **PI 40:4** | **-18.1** |
| **PS 42:3** | **15.4** | **PI 40:3** | **-8.5** |
| **PS 42:2** | **17.8** | ***Glycerophosphoserines*** |  |
| **PS 42:1** | **20.3** | **PS O-36:4 or PS P-36:3** | **-6.7** |
|  |  | **PS 38:4** | **-0.5** |
|  |  | **PS O-39:0** | **-0.5** |
|  |  | **PS O-40:4 or PS P-40:3** | **-12.3** |
|  |  | **PS 39:2** | **-4.9** |

**Supplemental Table 4.** Lipid and metabolites identified by SAM as differentially expressed in human patient samples belonging to low- and high-FABP4-expression groups based on DESI-MS imaging data analysis performed in the negative ion mode. Positive SAM scores represent higher relative abundance in samples with low-FABP4-expression, negative SAM scores represent higher relative abundance in samples with high-FABP4-expression. Tentative molecular formulas and chemical identification were attributed by high mass accuracy/high mass resolution and tandem MS analyses and are provided in supplemental Table 2.

| **HUMAN SAMPLES – MOLECULAR FORMULAS AND ATTRIBUTIONS** | | | | | | | |
| --- | --- | --- | --- | --- | --- | --- | --- |
| **Attribution** | **Molecular Formula** | **Detected *m/z*** | **Mass error (ppm)** | **Attribution** | **Molecular Formula** | **Detected *m/z*** | **Mass error (ppm)** |
| ***Metabolites*** |  |  |  | ***Ceramides*** |  |  |  |
| **Fumarate** | C_47_H_84_O_13_P | 115.004 | -1.7 | **Cer d32:1** | C_32_H_63_NO_3_Cl | 544.452 | 0.9 |
| **Acetyl-glycine** | C_4_H_6_NO_3_ | 116.035 | 1.7 | **Cer d16:1/17:0** | C_33_H_65_NO_3_Cl | 558.466 | 0.7 |
| **Succinate** | C_4_H_5_O_4_ | 117.020 | -1.7 | **Cer d34:2** | C_34_H_65_NO_3_Cl | 570.466 | 1.8 |
| **2-Hydroxy-3-methylbutyric acid** | C_5_H_9_O_3_ | 117.056 | -1.7 | **Cer d34:1** | C_34_H_67_NO_3_Cl | 572.481 | 0.5 |
| **Taurine** | C_2_H_6_NO_3_S | 124.008 | -0.8 | **Cer d18:16:0** | C_34_H_69_NO_3_Cl | 574.496 | 1.6 |
| **Pyroglutamate** | C_5_H_6_NO_3_ | 128.036 | -1.6 | **Cer d36:2** | C_36_H_69_NO_3_Cl | 598.496 | -1.7 |
| **Aspartate** | C_4_H_6_NO_4_ | 132.031 | -2.3 | **Cer d36:1** | C_36_H_71_NO_3_Cl | 600.512 | -2.4 |
| **3-Hydroxypicolinic acid** | C_6_H_4_NO_3_ | 138.020 | -0.7 | **Cer m18:1/22:0** | C_40_H_79_NO_2_Cl | 640.580 | 1.4 |
| **Glutamine** | C_5_H_9_N_2_O_3_ | 145.062 | -1.4 | **Cer d38:1** | C_40_H_79_NO_3_Cl | 656.575 | -0.1 |
| **Glutamic acid** | C_5_H_8_NO_4_ | 146.046 | 0.7 | **Cer m18:1/24:1** | C_42_H_81_NO_2_Cl | 666.597 | 2.1 |
| **2-Hydroxyglutarate** | C_5_H_7_O_5_ | 147.030 | -2.0 | **Cer d41:2** | C_41_H_79_NO_3_Cl | 668.577 | -0.4 |
| **Xanthine** | C_5_H_3_N_4_O_2_ | 151.026 | -1.3 | **Cer m42:1** | C_42_H_83_NO_2_Cl | 668.611 | 1.3 |
| **N-acetylaspartic acid** | C_6_H_8_NO_5_ | 174.041 | 0.1 | **Cer d18:1/23:0** | C_41_H_81_NO_2_Cl | 670.590 | 2.4 |
| **Ascorbic acid** | C_6_H_7_O_6_ | 175.025 | 2.3 | **Cer d40:0(2OH)** | C_40_H_81_NO_4_Cl | 674.587 | -2.1 |
| **Gluconic acid** | C_6_H_11_O_7_ | 195.051 | 0.5 | **Cer d42:0** | C_42_H_81_NO_3_Cl | 682.590 | 1.3 |
| **Phosphatidic acid** | C_6_H_8_O_7_Cl | 226.996 | 0.9 | **Cer d42:1** | C_42_H_83_NO_3_Cl | 684.607 | 1.0 |
| **Glycerophosphoglycerol** | C_6_H_14_O_8_P | 245.043 | -0.8 | **Cer d42:1** | C_42_H_83_NO_3_Cl | 684.607 | 0.1 |
| **Glutathione** | C_10_H_82_N_3_O_6_S | 306.076 | 2.6 | **Cer d42:0** | C_42_H_85_NO_3_Cl | 686.622 | 0.3 |
| ***Fatty Acids*** |  |  |  | **PE-Cer d36:1** | C_38_H_76_N_2_O_6_P | 687.545 | -0.4 |
| **FA 8:0** | C_8_H_15_O_2_ | 143.108 | 0.7 | **Cer d18:1/25:0** | C_43_H_85_NO_3_Cl | 698.622 | 0.3 |
| **FA 9:0** | C_9_H_17_O_2_ | 157.124 | -0.6 | **Cer d18:1/26:1** | C_44_H_85_NO_3_Cl | 710.626 | 4.9 |
| **FA 14:1** | C_14_H_25_O_2_ | 225.186 | -0.9 | **Cer d18:1/26:0** | C_44_H_87_NO_3_Cl | 712.637 | 1.5 |
| **FA 15:0** | C_15_H_29_O_2_ | 241.218 | 4.1 | **PE - Cer 36:3** | C_38_H_73_N_2_O_6_PCl | 719.488 | 2.6 |
| **FA 16:1** | C_16_H_29_O_2_ | 253.217 | -0.8 | **GlcCer d34:1** | C_40_H_77_NO_8_Cl | 734.532 | 2.7 |
| **FA 17:0** | C_17_H_33_O_2_ | 269.248 | 0.7 | **PE-Cer d37:1** | C_39_H_79_N_2_O_6_PCl | 737.536 | 1.5 |
| **FA hydroxy 16:0** | C_16_H_31_O_3_ | 271.228 | 0.4 | ***Cardiolipins*** |  |  |  |
| **FA 18:3** | C_18_H_29_O_2_ | 277.217 | 0.7 | **CL 68:5** | C_77_H_138_O_17_P_2_ | 698.472 | 0.9 |
| **FA 18:2** | C_18_H_31_O_2_ | 279.233 | 1.1 | **CL 70:7** | C_79_H_138_O_17_P_2_ | 710.470 | 1.8 |
| **FA 18:1** | C_18_H_32_O_2_ | 281.248 | 0.7 | **CL 70:6** | C_79_H_140_O_17_P_2_ | 711.477 | 3.0 |
| **FA 18:0** | C_18_H_35_O_2_ | 283.264 | 1.1 | **CL 70:5** | C_79_H_142_O_17_P_2_ | 712.484 | 4.2 |
| **FA 20:5** | C_20_H_29_O_2_ | 301.217 | 0.7 | **CL 72:8** | C_81_H_140_O_17_P_2_ | 723.479 | 0.4 |
| **FA 20:4** | C_20_H_31_O_2_ | 303.233 | 1.0 | **CL 72:7** | C_81_H_142_O_17_P_2_ | 724.484 | 3.6 |
| **FA 20:3** | C_20_H_33_O_2_ | 305.248 | -1.0 | **CL 72:6** | C_81_H_144_O_17_P_2_ | 725.494 | 0.6 |
| **FA 20:2** | C_20_H_35_O_2_ | 307.264 | -1.6 | **CL 72:4** | C_81_H_148_O_17_P_2_ | 727.507 | -1.9 |
| **FA 20:1** | C_20_H_37_O_2_ | 309.280 | 2.3 | **CL 74:10** | C_83_H_140_O_17_P_2_ | 735.478 | 1.2 |
| **FA 20:0** | C_20_H_39_O_2_ | 311.295 | -1.3 | **CL 74:9** | C_83_H_142_O_17_P_2_ | 736.485 | 2.0 |
| **FA 18:2** | C_18_H_32_O_2_Cl | 315.209 | 1.3 | **CL 74:8** | C_83_H_144_O_17_P_2_ | 737.492 | 3.3 |
| **FA 18:1** | C_18_H_34_O_2_Cl | 317.225 | 1.3 | **CL 74:7** | C_83_H_146_O_17_P_2_ | 738.501 | 1.1 |
| **FA hydroxy 20:4** | C_20_H_31_O_3_ | 319.228 | -1.6 | **CL 76:10** | C_85_H_144_O_17_P_2_ | 749.492 | 2.8 |
| **FA 18:0** | C_18_H_36_O_2_Cl | 319.241 | 0.6 | **CL 76:9** | C_85_H_146_O_17_P_2_ | 750.504 | 2.9 |
| **FA hydroxy 20:3** | C_20_H_33_O_3_ | 321.243 | 2.5 | **CL 76:8** | C_85_H_148_O_17_P_2_ | 751.509 | 2.0 |
| **FA 22:6** | C_22_H_31_O_2_ | 327.233 | 1.2 | **CL 76:7** | C_85_H_150_O_17_P_2_ | 752.516 | 2.5 |
| **FA 22:5** | C_22_H_33_O_2_ | 329.248 | 1.2 | **CL 36:4** | C_45_H_82_O_15_P_2_Cl | 959.482 | 2.4 |
| **FA 22:4** | C_22_H_35_O_2_ | 331.264 | 1.2 | ***Glycerophosphoinositols*** | |  |  |
| **FA 22:3** | C_22_H_37_O_2_ | 333.279 | 1.5 | **LysoPI (16:0/0:0)** | C_25_H_48_O_12_P | 571.290 | 1.8 |
| **FA 20:4** | C_20_H_32_O_2_Cl | 339.209 | 0.9 | **PI (32:1)** | C_41_H_76_O_13_P | 807.502 | 1.6 |
| **FA 22:0** | C_22_H_43_O_2_ | 339.326 | 1.5 | **PI (32:0)** | C_41_H_78_O_13_P | 809.514 | 5.6 |
| **FA hydroxy 22:6** | C_22_H_31_O_3_ | 343.230 | -5.0 | **PI (34:2)** | C_43_H_78_O_13_P | 833.517 | 2.4 |
| **FA 23:1** | C_23_H_43_O_2_ | 351.326 | 2.3 | **PI (34:1)** | C_43_H_80_O_13_P | 835.532 | 2.5 |
| **FA 23:0** | C_23_H_45_O_2_ | 353.342 | 1.4 | **PI (O-23:0)** | C_42_H_83_O_12_PCl | 845.532 | 0.2 |
| **FA 24:5** | C_24_H_37_O_2_ | 357.280 | 1.1 | **PI (25:1)** | C_44_H_82_O_13_P | 849.552 | -2.4 |
| **FA 24:4** | C_24_H_39_O_2_ | 359.295 | 0.8 | **PI (36:4)** | C_45_H_78_O_13_P | 857.517 | 1.6 |
| **FA 24:3** | C_24_H_41_O_2_ | 361.311 | 1.7 | **PI (36:3)** | C_45_H_80_O_13_P | 859.535 | -0.6 |
| **FA 24:1** | C_24_H_45_O_2_ | 365.342 | 1.4 | **PI (36:2)** | C_45_H_82_O_13_P | 861.549 | 1.5 |
| **FA 22:4** | C_22_H_36_O_2_Cl | 367.242 | -1.9 | **PI (36:1)** | C_45_H_84_O_13_P | 863.565 | 2.8 |
| **FA 24:0** | C_24_H_47_O_2_ | 367.358 | 1.1 | **PI (37:3)** | C_46_H_82_O_13_P | 873.551 | -0.8 |
| **FA hydroxy 24:0** | C_24_H_47_O_3_ | 383.353 | 1.3 | **PI (38:6)** | C_47_H_78_O_13_P | 881.520 | 1.1 |
| **FA 26:3** | C_26_H_45_O_2_ | 389.342 | 0.8 | **PI (38:3)** | C_47_H_84_O_13_P | 887.565 | -0.2 |
| **FA 26:2** | C_26_H_47_O_2_ | 391.358 | 1.0 | **PI (40:6)** | C_49_H_82_O_13_P | 909.547 | -3.0 |
| **FA 26:1** | C_26_H_49_O_5_ | 393.373 | 1.0 | **PI (39:4)** | C_48_H_85_O_13_PCl | 935.544 | 2.0 |
| **FA 26:0** | C_26_H_51_O_2_ | 395.389 | 1.5 | ***Glycerophosphoinositols*** | |  |  |
| ***Glycerolipids*** |  |  |  | **LysoPI 15:0** | C_24_H_46_O_12_P | 557.273 | 0.5 |
| **MG 18:0** | C_21_H_40_O_4_Cl | 391.262 | 1.5 | **LysoPI O-16:0** | C_25_H_50_O_11_P | 557.309 | 0.9 |
| **MG 22:6** | C_25_H_38_O_4_Cl | 437.246 | 1.1 | **LysoPI 18:0** | C_27_H_52_O_12_P | 599.320 | 0.5 |
| **DG 24:0/0:0** | C_27_H_51_O_5_ | 455.376 | 4.0 | **Lyso PI 20:4** | C_29_H_48_O_12_P | 619.290 | -2.4 |
| **DG 32:1/0:0** | C_35_H_66_O_5_Cl | 601.459 | 1.7 | **PI O-33:2 or PI P-33:1** | C_42_H_79_O_12_PCl | 841.501 | -1.0 |
| **DG 32:0/0:0** | C_35_H_68_O_5_Cl | 603.476 | 0.5 | **PI P-18:0/17:2** | C_44_H_81_O_12_PCl | 867.516 | 0.2 |
| **DG 34:3/0:0** | C_37_H_66_O_5_Cl | 625.459 | 1.6 | **PI O-35:2 or PI P-35:1** | C_44_H_83_O_12_PCl | 869.531 | 0.5 |
| **DG 34:2/0:0** | C_37_H_68_O_5_Cl | 627.475 | 1.1 | **PI 37:4** | C_46_H_80_O_13_P | 871.534 | 3.3 |
| **DG 34:1/0:0** | C_37_H_70_O_5_Cl | 629.491 | 0.6 | **PI 38:6** | C_47_H_78_O_13_P | 881.516 | 3.1 |
| **DG 36:4/0:0** | C_39_H_68_O_5_Cl | 651.475 | 1.2 | **PI 38:5** | C_47_H_80_O_13_P | 883.533 | 1.1 |
| **DG 36:3/0:0** | C_39_H_70_O_5_Cl | 653.492 | 0.2 | **PI 38:4** | C_47_H_82_O_13_P | 885.548 | 1.8 |
| **DG 36:2/0:0** | C_39_H_72_O_5_Cl | 655.507 | 0.3 | **PI 40:4** | C_49_H_86_O_13_P | 913.579 | 2.1 |
| **DG 38:6/0:0** | C_41_H_68_O_5_Cl | 675.475 | 1.3 | **PI 40:3** | C_49_H_88_O_13_P | 915.595 | 1.7 |
| **DG 38:5/0:0** | C_41_H_70_O_5_Cl | 677.493 | -2.2 | ***Glycerophosphoserines*** | | | |
| **DG 38:4/0:0** | C_41_H_72_O_5_Cl | 679.509 | -2.2 | **PS P-34:1** | C_40_H_75_NO_9_P | 744.518 | 1.1 |
| **DG 40:7/0:0** | C_43_H_70_O_5_Cl | 701.489 | 3.6 | **PS 16:0/18:1** | C_40_H_75_NO_10_P | 760.515 | 2.0 |
| **DG 40:6/0:0** | C_43_H_72_O_5_Cl | 703.506 | 1.6 | **PS O-36:4 or PS P-36:3** | C_42_H_75_NO_9_P | 768.521 | -3.4 |
| **DG 40:5/0:0** | C_43_H_74_O_5_Cl | 705.523 | 1.1 | **PS P-36:2 or PS O-36:3** | C_42_H_77_NO_9_P | 770.532 | 2.2 |
| ***Glycerophosphoethanolamines*** | |  |  | **PS O-36:2 or PS P-36:1** | C_42_H_79_NO_9_P | 772.549 | 1.0 |
| **LysoPE P-16:0** | C_21_H_43_NO_6_P | 436.284 | -2.3 | **PS 36:3** | C_42_H_75_NO_10_P | 784.513 | 0.1 |
| **LysoPE O-18:1** | C_23_H_47_NO_6_P | 464.316 | -2.8 | **PS 36:2** | C_42_H_77_NO_10_P | 786.528 | 1.5 |
| **LysoPE 18:1** | C_23_H_45_NO_7_P | 478.295 | -2.5 | **PS 18:0/18:1** | C_42_H_79_NO_10_P | 788.546 | -2.4 |
| **LysoPE 18:0** | C_23_H_47_NO_7_P | 480.311 | -2.7 | **PS O-38:4 or P-38:3** | C_44_H_79_NO_9_P | 796.547 | 3.9 |
| **LysoPE 20:4** | C_23_H_43_NO_7_P | 500.280 | -2.6 | **PS 38:4** | C_44_H_77_NO_10_P | 810.528 | 1.5 |
| **PE 18:1/1:0** | C_24_H_45_NO_8_P | 506.288 | 1.2 | **PS 38:3** | C_44_H_79_NO_10_P | 812.544 | 1.2 |
| **PE 20:4/1:0** | C_26_H_43_NO_8_P | 528.273 | -0.8 | **PS 38:2** | C_44_H_81_NO_10_P | 814.558 | 3.3 |
| **PE 20:3/1:0** | C_26_H_45_NO_8_P | 530.291 | -4.5 | **PS 38:1** | C_44_H_83_NO_10_P | 816.574 | 1.8 |
| **PE O-34:3 or PE P-34:2** | C_39_H_73_NO_7_P | 698.515 | 3.3 | **PS O-39:0** | C_45_H_89_NO_9_P | 818.625 | 4.3 |
| **PE O-34:2 or PE P-34:1** | C_39_H_75_NO_7_P | 700.527 | 2.1 | **PS O-40:4 or PS P-40:3** | C_46_H_83_NO_9_P | 824.581 | 0.1 |
| **PE 34:2** | C_39_H_73_NO_8_P | 714.505 | 3.8 | **PS 39:2** | C_45_H_83_NO_10_P | 828.572 | 4.3 |
| **PE 34:1** | C_39_H_75_NO_8_P | 716.522 | 2.1 | **PS 40:6** | C_46_H_77_NO_10_P | 834.527 | 2.4 |
| **PE 34:0** | C_23_H_47_NO_6_P | 718.538 | 1.9 | **PS 40:2** | C_46_H_85_NO_10_P | 842.591 | 1.3 |
| **PE P-18:0/18:4** | C_41_H_73_NO_7_Cl | 722.512 | 1.9 | **PS 40:1** | C_46_H_87_NO_10_P | 844.606 | 2.0 |
| **PE O-36:3 or P-36:2** | C_41_H_77_NO_7_P | 726.545 | 0.8 | **PS 42:3** | C_48_H_87_NO_10_P | 868.603 | -5.1 |
| **PE 35:1** | C_40_H_77_NO_8_P | 730.537 | 3.3 | **PS 42:2** | C_48_H_89_NO_10_P | 870.622 | -1.1 |
| **PE 36:3** | C_41_H_75_NO_8_P | 740.520 | 3.8 | **PS 42:1** | C_48_H_91_NO_10_P | 872.637 | -1.9 |
| **PE 36:2** | C_41_H_77_NO_8_P | 742.538 | 1.9 | ***Glycerophosphoglycerols*** | |  |  |
| **PE O-38:5 or PE P-38:4** | C_43_H_77_NO_7_P | 750.543 | -1.2 | **LysoPG 16:0** | C_22_H_44_O_9_P | 483.273 | -2.1 |
| **PE 38:5** | C_43_H_75_NO_8_P | 764.522 | -3.2 | **LysoPG 18:2** | C_24_H_44_O_9_P | 507.274 | -3.1 |
| **PE 38:4** | C_43_H_77_NO_8_P | 766.538 | 6.2 | **LysoPG 18:1** | C_24_H_46_O_9_P | 509.288 | -2.9 |
| **PE 38:3** | C_43_H_79_NO_8_P | 768.557 | -3.3 | **LysoPG 22:6** | C_28_H_44_O_9_P | 555.274 | -0.7 |
| **PE 38:2** | C_43_H_81_NO_8_P | 770.566 | 1.2 | **PG 34:3** | C_40_H_72_O_10_P | 743.489 | -2.8 |
| **PE 38:1** | C_43_H_83_NO_8_P | 772.585 | 1.9 | **PG 34:2** | C_40_H_74_O_10_P | 745.502 | 1.5 |
| **PE 39:6** | C_44_H_75_NO_8_P | 776.522 | 1.8 | **PG 16:0/18:1** | C_40_H_76_O_10_P | 747.520 | 1.6 |
| **PE 39:5** | C_44_H_77_NO_8_P | 778.538 | -1.0 | **PG 36:4** | C_42_H_74_O_10_P | 769.501 | 2.3 |
| **PE O-40:5 or PE P-40:4** | C_45_H_81_NO_7_P | 778.576 | 0.5 | **PG 18:2/18:1** | C_42_H_76_O_10_P | 771.520 | -2.5 |
| **PE 40:5** | C_45_H_79_NO_8_P | 792.555 | -1.6 | **PG 18:1/18:1** | C_42_H_78_O_10_P | 773.533 | 0.9 |
| **PE 37:1** | C_42_H_82_NO_8_PCl | 794.549 | -0.8 | **PG 18:0/18:1** | C_42_H_80_O_10_P | 775.548 | 1.7 |
| **PE 40:4** | C_45_H_81_NO_8_P | 794.571 | 0.7 | **PG 18:0/18:0** | C_42_H_82_O_10_P | 777.565 | 0.3 |
| **PE 39:4** | C_44_H_80_NO_8_PCl | 816.531 | 0.7 | **PG 38:6** | C_44_H_74_O_10_P | 793.501 | 1.9 |
| **PE 39:2** | C_44_H_84_NO_8_PCl | 820.562 | 2.9 | **PG 38:5** | C_44_H_76_O_10_P | 795.515 | 4.7 |
| **PE 39:1** | C_44_H_86_NO_8_PCl | 822.576 | -4.0 | **PG 38:4** | C_44_H_78_O_10_P | 797.531 | 3.1 |
| **PE 41:6** | C_46_H_80_NO_8_PCl | 840.535 | -2.7 | **PG 38:3** | C_44_H_80_O_10_P | 799.547 | 3.5 |
| **PE 41:5** | C_46_H_82_NO_8_PCl | 842.550 | 1.1 | **PG 38:2** | C_44_H_82_O_10_P | 801.563 | 2.1 |
| **PE 41:4** | C_46_H_84_NO_8_PCl | 844.562 | -1.4 | **PG 40:8** | C_46_H_74_O_10_P | 817.501 | 1.7 |
| **PE 43:6** | C_48_H_84_NO_8_PCl | 868.564 | -3.4 | **PG 40:7** | C_46_H_76_O_10_P | 819.516 | 2.7 |
| **PE 43:2** | C_48_H_92_NO_8_PCl | 876.629 | 0.8 | **PG 40:6** | C_46_H_78_O_10_P | 821.531 | 3.5 |
| ***Monoacylglycerophosphates*** | |  |  | **PG 40:5** | C_46_H_80_O_10_P | 823.548 | 1.8 |
| **PA 32:0** | C_35_H_68_O_8_P | 647.468 | 3.2 | **PG 42:7** | C_48_H_80_O_10_P | 847.546 | 3.8 |
| **PA 24:2** | C_37_H_68_O_8_P | 671.464 | 2.2 | **PG 22:6/22:6** | C_50_H_74_O_10_P | 865.500 | 3.4 |
| **PA 24:1** | C_37_H_70_O_8_P | 673.481 | 1.6 |  |  |  |  |
| **PA 36:4** | C_39_H_68_O_8_P | 695.465 | 1.6 |  |  |  |  |
| **PA 36:3** | C_39_H_70_O_8_P | 697.480 | 1.9 |  |  |  |  |
| **PA 36:1** | C_39_H_74_O_8_P | 701.512 | 1.0 |  |  |  |  |
| **PA O-38:2 or PA P-38:1** | C_41_H_79_O_7_PCl | 749.528 | 1.3 |  |  |  |  |

**Supplementary Table 5.** Lipid and metabolite molecular attributions for *m/z* values selected by SAM as differentially expressed in human patient samples belonging to low- and high-FABP4-expression groups based on DESI-MS imaging data analysis performed in the negative ion mode. Tentative molecular formulas and chemical identification were attributed by high mass accuracy/high mass resolution and tandem MS analyses.

| **siFABP4 - LOW FABP4 EXPRESSION** | | **CONTROL - HIGH FABP4 EXPRESSION** | | | |
| --- | --- | --- | --- | --- | --- |
| **Attribution** | **SAM Score** | **Attribution** | **SAM Score** | **Attribution** | **SAM Score** |
| **Metabolites** |  | **Metabolites** |  | **PE O-36:3 or P-36:2** | **-5.2** |
| **Succinate** | **14.4** | **Uracil** | **-7.6** | **PE 36:3** | **-9.7** |
| **Glutathione** | **2.4** | **Taurine** | **-5.8** | **PE 36:2** | **-14.6** |
| **Methymycin** | **3.6** | **Xanthine** | **-6.7** | **PE 36:1** | **-10.9** |
| **Glycerolipids** |  | **Ascorbic acid** | **-2.4** | **PE O-38:5 or PE P-38:4** | **-22.4** |
| **DG 32:1/0:0** | **5.5** | **Citrate** | **-10.0** | **PE 38:5** | **-11.7** |
| **DG 34:2/0:0** | **4.2** | **Lauric Acid** | **-11.0** | **PE 38:4** | **-24.9** |
| **DG 34:1/0:0** | **5.8** | **Glycerophosphoethanolamine** | **-12.6** | **PE 40:6** | **-15.8** |
| **DG 36:4/0:0** | **1.3** | **Glucose** | **-4.5** | **PE 40:5** | **-6.4** |
| **DG 36:1/0:0** | **3.1** | **Norselic acid A** | **-11.0** | **PE 40:4** | **-18.5** |
| **DG 38:6/0:0** | **4.1** | **Fatty Acids** |  | **Glycerophosphoglycerols** | |
| **DG 38:5/0:0** | **5.2** | **FA 8:0** | **-10.2** | **LysoPG 18:2** | **-8.5** |
| **DG 38:4/0:0** | **3.4** | **FA 14:0** | **-15.8** | **LysoPG 18:1** | **-4.0** |
| **DG 40:8/0:0** | **4.6** | **FA 15:0** | **-13.2** | **LysoPG 22:6** | **-7.8** |
| **DG 40:7/0:0** | **3.2** | **FA 16:2** | **-9.2** | **PG 16:0/18:1** | **-8.0** |
| **DG 40:7/0:0** | **3.1** | **FA 16:1** | **-16.7** | **PG 36:4** | **-12.5** |
| **Glycerophosphoethanolamines** | | **FA 16:0** | **-22.4** | **PG 18:2/18:1** | **-4.6** |
| **PE 18:1** | **2.4** | **FA 18:3** | **-14.7** | **PG 18:1/18:1** | **-6.0** |
| **PE 36:2** | **2.0** | **FA 18:2** | **-22.8** | **PG 38:2** | **-10.7** |
| **PE 38:6** | **4.5** | **FA 18:1** | **-22.7** | **PG 40:8** | **-6.7** |
| **PE 37:1** | **2.4** | **FA 18:0** | **-21.9** | **Monoacylglycerophosphates** | |
| **PE 38:6** | **4.5** | **FA hydroxy 18:2** | **-9.0** | **PA 32:0** | **-5.4** |
| **PE 39:2** | **2.9** | **FA 19:1** | **-19.2** | **PA 24:2** | **-5.7** |
| ***Glycerophosphoglycerols*** | | **FA 19:0** | **-16.8** | **PA 36:4** | **-8.4** |
| **PG 22:1** | **3.0** | **FA 20:5** | **-7.6** | **PA 36:3** | **-9.1** |
| **PG 34:1** | **2.7** | **FA 20:4** | **-22.2** | **PA 36:2** | **-6.6** |
| **PG P-26:2 or PG O-36:3** | **8.1** | **FA 20:3** | **-20.1** | **Cardiolipins** |  |
| **PG P-36:1 or PG O-36:2** | **3.1** | **FA 20:2** | **-21.5** | **CL 72:8** | **-7.7** |
| **PG 18:0/18:1** | **4.6** | **FA 20:1** | **-21.2** | **CL 72:7** | **-8.5** |
| **PG 18:0/18:0** | **5.3** | **FA 20:0** | **-15.4** | **CL 72:4** | **-4.5** |
| **PG 38:7** | **2.0** | **FA 18:2** | **-6.6** | **CL 74:10** | **-6.7** |
| **PG 38:5** | **2.1** | **FA 18:1** | **-6.1** | **CL 74:9** | **-8.4** |
| **PG 42:9** | **5.5** | **FA hydroxy 20:4** | **-6.3** | **CL 74:8** | **-16.3** |
| ***Ceramides*** |  | **FA 22:6** | **-16.9** | **CL 74:7** | **-18.1** |
| **Cer d32:1** | **2.9** | **FA 22:5** | **-17.2** | **CL 74:6** | **-16.2** |
| **Cer 34:2** | **5.9** | **FA 22:4** | **-20.9** | **CL 79:10** | **-8.1** |
| **Cer 34:1** | **5.5** | **FA 22:3** | **-17.6** | **CL 79:9** | **-5.9** |
| **Cer d18/16:0** | **3.9** | **FA 22:2** | **-18.5** | **CL 79:8** | **-9.6** |
| **Cer m18:1/22:0** | **3.3** | **FA 22:1** | **-15.5** | **CL 79:7** | **-5.8** |
| **Cer d40:2** | **5.1** | **FA 20:4** | **-6.1** | **Glycerophosphoinositols** | |
| **Cer d40:2** | **2.6** | **FA 22:0** | **-9.5** | **LysoPI 18:0** | **-13.7** |
| **Cer m42:1** | **6.5** | **FA 24:6** | **-17.0** | **LysoPI 20:4** | **-7.5** |
| **Cer d42:1** | **9.3** | **FA 24:5** | **-16.7** | **PI 36:4** | **-12.7** |
| **Cer d42:0** | **6.9** | **FA 24:4** | **-21.3** | **PI 36:3** | **-8.4** |
| **Cer m44:1** | **5.4** | **FA 24:3** | **-17.2** | **PI 36:2** | **-9.9** |
| **Cer d18:1/26:1** | **3.5** | **FA 24:2** | **-17.7** | **PI 37:4** | **-7.5** |
| **Cer d18:1/26:0** | **7.6** | **FA 24:1** | **-15.1** | **PI 38:5** | **-10.4** |
| **GlcCer d34:2** | **4.8** | **FA 22:4** | **-7.1** | **PI 38:4** | **-18.0** |
| **GlcCer d34:1** | **6.5** | **FA 24:0** | **-6.1** | **PI 38:3** | **-13.4** |
| ***Cardiolipins*** |  | **FA 25:1** | **-5.2** | **PI 39:4** | **-8.6** |
| **CL 70:7** | **4.1** | **FA methyl 24:0** | **-7.9** | **PI 40:6** | **-10.1** |
| **CL 70:6** | **2.8** | **FA 26:5** | **-17.0** | **PI 40:5** | **-11.6** |
| **CL 74:10** | **0.6** | **FA 26:4** | **-20.9** | **PI 40:4** | **-19.7** |
| **CL 74:9** | **1.6** | **FA 26:3** | **-16.2** | **Glycerophosphoserines** | |
| ***Glycerophosphoinositols*** | | **FA 26:2** | **-17.6** | **PS 36:4** | **-7.0** |
| **PI O-31:1 or PI P-31:0** | **2.0** | **FA 26:1** | **-12.3** | **PS 36:3** | **-4.5** |
| ***Glycerophosphoserines*** | | **FA 28:2** | **-14.2** | **PS 36:2** | **-13.6** |
| **PS P-33:0** | **5.0** | **FA 26:0** | **-6.2** | **PS 18:0/18:1** | **-10.4** |
| **PS P-36:2 or PS O-36:3** | **3.8** | **Glycerolipids** |  | **PS 38:4** | **-21.5** |
| **PS O-36:2 or PS P-36:1** | **2.1** | **MG 18:0/0:0** | **-11.4** | **PS 38:3** | **-13.0** |
| **PS 18:0/18:1** | **4.7** | **DG 36:3/0:0** | **-3.1** | **PS 38:2** | **-8.5** |
| **PS O-39:0** | **2.0** | **DG 36:2/0:0** | **-2.5** | **PS 39:4** | **-9.4** |
| **PS 40:2** | **2.7** | **Glycerophosphoethanolamines** |  | **PS 39:3** | **-4.4** |
| **PS 40:1** | **3.8** | **LysoPE 16:0** | **-10.5** | **PS 40:6** | **-12.2** |
| **PS O-41:0** | **9.7** | **LysoPE 18:0** | **-13.3** | **PS 40:4** | **-20.6** |
| **PS 42:2** | **4.3** | **PE 34:2** | **-6.2** | **PS 22:6/19:0** | **-10.3** |
| **PS 42:1** | **3.5** | **PE P-18:0/18:4** | **-18.2** | **PS 41:4** | **-7.7** |

**Supplementary Table 6.** SAM scores assigned to lipid and metabolites selected as differentially expressed in six mouse model samples (3 siFABP4, 3 siControl) based on DESI-MS imaging data analysis performed in the negative ion mode. Positive SAM scores represent higher in low-FABP4-expression (siFABP4 samples), negative SAM scores represent higher in high-FABP4-expression (siControl samples). Tentative molecular formulas and chemical identification were attributed by high mass accuracy/high mass resolution and tandem MS analyses and are included in Supplemental Table 4.

| **MICE SAMPLES - MOLECULAR FORMULAS AND ATTRIBUTIONS** | | | | | | | |
| --- | --- | --- | --- | --- | --- | --- | --- |
| **Attribution** | **Molecular Formula** | **Detected *m/z*** | **Mass error (ppm)** | **Attribution** | **Molecular Formula** | **Detected *m/z*** | **Mass error (ppm)** |
| **Metabolites** |  |  |  | **Glycerophosphoethanolamines** | |  |  |
| **Uracil** | C_4_H_3_O_2_N_2_ | 111.020 | 0.9 | **LysoPE 16:0** | C_21_H_43_NO_6_P | 436.282 | 2.8 |
| **Succinate** | C_4_H_5_O_4_ | 117.019 | 0.6 | **LysoPE 18:0** | C_23_H_47_NO_7_P | 480.308 | 2.5 |
| **Taurine** | C_2_H_6_NO_3_S | 124.007 | 0.8 | **PE 18:1** | C_24_H_45_NO_8_P | 506.288 | 2.4 |
| **Xanthine** | C_5_H_3_O_2_N_4_ | 151.026 | -0.7 | **PE 34:2** | C_39_H_73_NO_8_P | 714.506 | 2.4 |
| **Ascorbic acid** | C_6_H_7_O_6_ | 175.025 | -1.7 | **PE P-18:0/18:4** | C_41_H_73_NO_7_P | 722.511 | 2.6 |
| **Citrate** | C_6_H_7_O_7_ | 191.019 | -2.1 | **PE O-36:3 or P-36:2** | C_41_H_77_NO_7_P | 726.542 | -3.6 |
| **Lauric Acid** | C_12_H_23_O_2_ | 199.170 | -2.5 | **PE 36:3** | C_41_H_75_NO_8_P | 740.522 | 2.6 |
| **Glycerophosphoethanolamine** | C_5_H_13_NO_6_P | 214.048 | -2.3 | **PE 36:2** | C_41_H_77_NO_8_P | 742.537 | 2.7 |
| **Glucose** | C_6_H_12_O_6_Cl | 215.032 | -2.3 | **PE 36:2** | C_41_H_77_NO_8_P | 742.538 | 1.9 |
| **Glutathione** | C_10_H_16_N_3_O_6_S | 306.076 | -2.9 | **PE 36:1** | C_41_H_79_NO_8_P | 744.552 | 3.9 |
| **Norselic acid A** | C_29_H_39_O_2_ | 451.286 | -1.1 | **PE O-38:5 or PE P-38:4** | C_43_H_77_NO_7_P | 750.542 | 2.7 |
| **Methymycin** | C_25_H_43_NO_7_Cl | 504.272 | -2.8 | **PE 38:6** | C_43_H_73_NO_8_P | 762.504 | 5.1 |
| **Fatty Acids** |  |  |  | **PE 38:6** | C_43_H_73_NO_8_P | 762.504 | 5.1 |
| **FA 8:0** | C_8_H_15_O_2_ | 143.108 | -1.4 | **PE 38:5** | C_43_H_75_NO_8_P | 764.522 | 2.5 |
| **FA 14:0** | C_14_H_27_O_2_ | 227.201 | -2.6 | **PE 38:4** | C_43_H_77_NO_8_P | 766.537 | 2.3 |
| **FA 15:0** | C_15_H_29_O_2_ | 241.217 | -2.9 | **PE 40:6** | C_45_H_77_NO_8_P | 790.540 | -1.4 |
| **FA 16:2** | C_16_H_27_O_2_ | 251.201 | -3.6 | **PE 40:5** | C_45_H_79_NO_8_P | 792.555 | -0.1 |
| **FA 16:1** | C_16_H_29_O_2_ | 253.217 | -2.4 | **PE 37:1** | C_42_H_82_NO_8_PCl | 794.544 | 3.5 |
| **FA 16:0** | C_16_H_31_O_2_ | 255.232 | -2.4 | **PE 40:4** | C_45_H_81_NO_8_P | 794.571 | -0.3 |
| **FA 18:3** | C_18_H_29_O_2_ | 277.217 | -2.2 | **PE 39:2** | C_44_H_84_NO_8_PCl | 820.560 | 4.1 |
| **FA 18:2** | C_18_H_31_O_2_ | 279.232 | -2.1 | ***Glycerophosphoglycerols*** | |  |  |
| **FA 18:1** | C_18_H_33_O_2_ | 281.248 | -2.1 | **LysoPG 18:2** | C_24_H_44_O_9_P | 507.272 | 2.0 |
| **FA 18:0** | C_18_H_35_O_2_ | 283.264 | -2.1 | **LysoPG 18:1** | C_24_H_46_O_9_P | 509.288 | 1.8 |
| **FA hydroxy 18:2** | C_18_H_31_O_3_ | 295.227 | -2.7 | **LysoPG 22:6** | C_28_H_44_O_9_P | 555.272 | 1.4 |
| **FA 19:1** | C_19_H_35_O_2_ | 295.264 | -2.7 | **PG 22:1** | C_38_H_72_O_10_P | 719.486 | 1.7 |
| **FA 19:0** | C_19_H_37_O_2_ | 297.279 | -2.4 | **PG 34:1** | C_40_H_72_O_10_P | 743.485 | 2.6 |
| **FA 20:5** | C_20_H_29_O_2_ | 301.217 | 2.7 | **PG 16:0/18:1** | C_40_H_76_O_10_P | 747.516 | 2.9 |
| **FA 20:4** | C_20_H_31_O_2_ | 303.232 | 2.6 | **PG P-26:2 or PG O-36:3** | C_42_H_78_O_9_P | 757.539 | 0.5 |
| **FA 20:3** | C_20_H_33_O_2_ | 305.248 | 2.9 | **PG P-36:1 or PG O-36:2** | C_42_H_80_O_9_P | 759.553 | 2.5 |
| **FA 20:2** | C_20_H_35_O_2_ | 307.263 | 2.9 | **PG 36:4** | C_42_H_74_O_10_P | 769.500 | 3.0 |
| **FA 20:1** | C_20_H_37_O_2_ | 309.279 | 2.6 | **PG 18:2/18:1** | C_42_H_76_O_10_P | 771.515 | 3.9 |
| **FA 20:0** | C_20_H_39_O_2_ | 311.295 | 3.2 | **PG 18:1/18:1** | C_42_H_78_O_10_P | 773.532 | 2.8 |
| **FA 18:2** | C_18_H_32_O_2_Cl | 315.209 | 2.9 | **PG 18:0/18:1** | C_42_H_80_O_10_P | 775.548 | 1.7 |
| **FA 18:1** | C_18_H_34_O_2_Cl | 317.225 | 2.5 | **PG 18:0/18:0** | C_42_H_82_O_10_P | 777.565 | 0.3 |
| **FA hydroxy 20:4** | C_20_H_31_O_3_ | 319.227 | 1.9 | **PG 38:7** | C_44_H_72_O_10_P | 791.485 | 2.7 |
| **FA 22:6** | C_22_H_31_O_2_ | 327.232 | 2.8 | **PG 38:5** | C_44_H_76_O_10_P | 795.515 | 4.7 |
| **FA 22:5** | C_22_H_33_O_2_ | 329.248 | 2.7 | **PG 38:2** | C_44_H_82_O_10_P | 801.564 | 1.5 |
| **FA 22:4** | C_22_H_35_O_2_ | 331.264 | 1.2 | **PG 40:8** | C_46_H_74_O_10_P | 817.500 | 2.6 |
| **FA 22:3** | C_22_H_37_O_2_ | 333.279 | 2.7 | **PG 42:9** | C_48_H_76_O_10_P | 843.515 | 4.3 |
| **FA 22:2** | C_22_H_39_O_2_ | 335.295 | 1.2 | ***Ceramides*** |  |  |  |
| **FA 22:1** | C_22_H_41_O_2_ | 337.310 | 3.0 | **Cer d32:1** | C_32_H_63_NO_3_Cl | 544.449 | 1.8 |
| **FA 20:4** | C_20_H_32_O_2_Cl | 339.209 | 2.4 | **Cer 34:2** | C_34_H_65_NO_3_Cl | 570.464 | 2.5 |
| **FA 22:0** | C_22_H_43_O_2_ | 339.326 | 3.2 | **Cer 34:1** | C_34_H_67_NO_3_Cl | 572.480 | 2.3 |
| **FA 24:6** | C_24_H_35_O_2_ | 355.263 | 2.5 | **Cer d18/16:0** | C_34_H_69_NO_3_Cl | 574.496 | 1.9 |
| **FA 24:5** | C_24_H_37_O_2_ | 357.279 | 2.8 | **Cer m18:1/22:0** | C_40_H_79_NO_2_Cl | 640.579 | 2.3 |
| **FA 24:4** | C_24_H_39_O_2_ | 359.295 | 2.5 | **Cer d40:2** | C_40_H_77_NO_3_Cl | 654.559 | 1.8 |
| **FA 24:3** | C_24_H_41_O_2_ | 361.310 | 2.5 | **Cer d40:2** | C_40_H_77_NO_3_Cl | 656.574 | 2.0 |
| **FA 24:2** | C_24_H_43_O_2_ | 363.326 | 1.9 | **Cer m42:1** | C_42_H_83_NO_2_Cl | 668.611 | 1.6 |
| **FA 24:1** | C_24_H_45_O_2_ | 365.342 | 2.5 | **Cer d42:1** | C_42_H_83_NO_3_Cl | 684.607 | 0.6 |
| **FA 22:4** | C_22_H_36_O_2_Cl | 367.242 | -3.3 | **Cer d42:0** | C_42_H_85_NO_3_Cl | 686.621 | 2.3 |
| **FA 24:0** | C_24_H_47_O_2_ | 367.358 | 1.1 | **Cer m44:1** | C_44_H_87_NO_2_Cl | 696.641 | 2.6 |
| **FA 25:1** | C_25_H_47_O_2_ | 379.357 | 2.4 | **Cer d18:1/26:1** | C_44_H_85_NO_3_Cl | 710.624 | 2.3 |
| **FA methyl 24:0** | C_25_H_49_O_2_ | 381.373 | 1.8 | **Cer d18:1/26:0** | C_44_H_87_NO_3_Cl | 712.638 | 0.1 |
| **FA 26:5** | C_26_H_41_O_2_ | 385.311 | 1.8 | **GlcCer d34:2** | C_50_H_95_NO_8_Cl | 872.676 | -0.6 |
| **FA 26:4** | C_26_H_43_O_2_ | 387.326 | 2.3 | **GlcCer d34:1** | C_50_H_97_NO_8_Cl | 874.687 | 4.5 |
| **FA 26:3** | C_26_H_45_O_2_ | 389.342 | 2.3 | ***Cardiolipins*** |  |  |  |
| **FA 26:2** | C_26_H_47_O_2_ | 391.357 | 2.3 | **CL 70:7** | C_79_H_138_O_17_P_2_ | 710.469 | 3.0 |
| **FA 26:1** | C_26_H_49_O_2_ | 393.373 | 1.0 | **CL 70:6** | C_79_H_140_O_17_P_2_ | 711.476 | 4.6 |
| **FA 28:2** | C_26_H_51_O_2_ | 395.388 | 2.8 | **CL 72:8** | C_81_H_140_O_17_P_2_ | 723.477 | -3.0 |
| **FA 26:0** | C_26_H_51_O_2_ | 395.389 | 2.5 | **CL 72:7** | C_81_H_142_O_17_P_2_ | 724.483 | 4.6 |
| **Glycerolipids** |  |  |  | **CL 72:4** | C_81_H_148_O_17_P_2_ | 727.507 | 4.3 |
| **MG 18:0/0:0** | C_21_H_40_O_4_Cl | 391.261 | 2.8 | **CL 74:10** | C_83_H_140_O_17_P_2_ | 735.476 | 4.1 |
| **DG 32:1/0:0** | C_35_H_66_O_5_Cl | 601.459 | 1.8 | **CL 74:10** | C_83_H_140_O_17_P_2_ | 735.478 | 1.2 |
| **DG 34:2/0:0** | C_37_H_68_O_5_Cl | 627.475 | -2.2 | **CL 74:9** | C_83_H_142_O_17_P_2_ | 736.485 | 2.7 |
| **DG 34:1/0:0** | C_37_H_70_O_5_Cl | 629.491 | -0.5 | **CL 74:9** | C_83_H_142_O_17_P_2_ | 736.485 | 2.0 |
| **DG 36:4/0:0** | C_39_H_68_O_5_Cl | 651.475 | 2.0 | **CL 74:8** | C_83_H_144_O_17_P_2_ | 737.492 | 3.1 |
| **DG 36:3/0:0** | C_39_H_70_O_5_Cl | 653.491 | 1.4 | **CL 74:7** | C_83_H_146_O_17_P_2_ | 738.502 | 0.1 |
| **DG 36:2/0:0** | C_39_H_72_O_5_Cl | 655.508 | -0.2 | **CL 74:6** | C_83_H_148_O_17_P_2_ | 739.507 | 3.7 |
| **DG 36:1/0:0** | C_39_H_74_O_5_Cl | 657.523 | 0.2 | **CL 79:10** | C_85_H_144_O_17_P_2_ | 749.492 | 2.8 |
| **DG 38:6/0:0** | C_41_H_68_O_5_Cl | 675.475 | 1.5 | **CL 79:9** | C_85_H_146_O_17_P_2_ | 750.503 | -0.4 |
| **DG 38:5/0:0** | C_41_H_70_O_5_Cl | 677.490 | 2.1 | **CL 79:8** | C_85_H_148_O_17_P_2_ | 751.508 | 3.2 |
| **DG 38:4/0:0** | C_41_H_72_O_5_Cl | 679.506 | 2.5 | **CL 79:7** | C_85_H_150_O_17_P_2_ | 752.518 | 0.1 |
| **DG 40:8/0:0** | C_43_H_68_O_5_Cl | 699.474 | 2.7 | ***Glycerophosphoserines*** | |  |  |
| **DG 40:7/0:0** | C_43_H_70_O_5_Cl | 701.490 | 2.3 | **PS P-33:0** | C_39_H_75_NO_9_P | 732.517 | 1.9 |
| **DG 40:7/0:0** | C_43_H_72_O_5_Cl | 703.506 | 1.6 | **PS P-36:2 or PS O-36:3** | C_42_H_77_NO_9_P | 770.532 | 2.2 |
| **Monoacylglycerophosphates** | |  |  | **PS O-36:2 or PS P-36:1** | C_42_H_79_NO_9_P | 772.549 | 1.0 |
| **PA 32:0** | C_35_H_68_O_8_P | 647.464 | -2.8 | **PS 36:4** | C_42_H_73_NO_10_P | 782.496 | 2.2 |
| **PA 24:2** | C_37_H_68_O_8_P | 671.464 | 2.4 | **PS 36:3** | C_42_H_75_NO_10_P | 784.512 | 1.9 |
| **PA 36:4** | C_39_H_68_O_8_P | 695.464 | 2.0 | **PS 36:2** | C_42_H_77_NO_10_P | 786.527 | 2.7 |
| **PA 36:3** | C_39_H_70_O_8_P | 697.480 | 1.4 | **PS 18:0/18:1** | C_42_H_79_NO_10_P | 788.543 | 2.3 |
| **PA 36:2** | C_39_H_72_O_8_P | 699.495 | 3.1 | **PS 18:0/18:1** | C_42_H_79_NO_10_P | 788.547 | -2.4 |
| ***Glycerophosphoinositols*** | |  |  | **PS 38:4** | C_44_H_77_NO_10_P | 810.527 | 2.7 |
| **LysoPI 18:0** | C_27_H_52_O_12_P | 599.319 | 2.2 | **PS 38:3** | C_44_H_79_NO_10_P | 812.542 | 3.8 |
| **LysoPI 20:4** | C_29_H_48_O_12_P | 619.287 | 3.4 | **PS 38:2** | C_44_H_81_NO_10_P | 814.558 | 3.3 |
| **PI O-31:1 or PI P-31:0** | C_40_H_77_O_12_PCl | 815.486 | -1.1 | **PS O-39:0** | C_45_H_89_NO_9_P | 818.626 | 2.7 |
| **PI 36:4** | C_45_H_78_O_13_P | 857.516 | 2.7 | **PS 39:4** | C_45_H_79_NO_10_P | 824.543 | 1.8 |
| **PI 36:3** | C_45_H_80_O_13_P | 859.531 | 3.5 | **PS 39:3** | C_45_H_81_NO_10_P | 826.563 | -2.9 |
| **PI 36:2** | C_45_H_82_O_13_P | 861.548 | 2.8 | **PS 40:6** | C_46_H_77_NO_10_P | 834.527 | 2.6 |
| **PI 37:4** | C_46_H_80_O_13_P | 871.533 | 1.1 | **PS 40:4** | C_46_H_81_NO_10_P | 838.558 | 3.1 |
| **PI 38:5** | C_47_H_80_O_13_P | 883.532 | 2.4 | **PS 40:2** | C_46_H_85_NO_10_P | 842.589 | 2.7 |
| **PI 38:4** | C_47_H_82_O_13_P | 885.547 | 2.8 | **PS 40:1** | C_46_H_87_NO_10_P | 844.606 | 2.0 |
| **PI 38:3** | C_47_H_84_O_13_P | 887.565 | -0.2 | **PS O-41:0** | C_47_H_93_NO_9_P | 846.658 | 1.3 |
| **PI 39:4** | C_48_H_84_O_13_P | 899.563 | 2.9 | **PS 22:6/19:0** | C_46_H_81_NO_10_P | 848.544 | 0.9 |
| **PI 40:6** | C_49_H_82_O_13_P | 909.548 | -2.1 | **PS 41:4** | C_47_H_83_NO_10_P | 852.573 | 3.3 |
| **PI 40:5** | C_49_H_84_O_13_P | 911.563 | -3.1 | **PS 42:2** | C_48_H_89_NO_10_P | 870.621 | 2.0 |
| **PI 40:4** | C_49_H_86_O_13_P | 913.578 | 3.2 | **PS 42:1** | C_48_H_91_NO_10_P | 872.636 | 3.3 |

**Supplementary Table 7.** Lipid and metabolite molecular attributions for *m/z* values selected by SAM as differentially expressed in siFABP4 and control mouse model samples based on DESI-MS imaging data analysis performed in the negative ion mode. Tentative molecular formulas and chemical identification were attributed by high mass accuracy/high mass resolution and tandem MS analyses.

| siRNA | Sequence |
| --- | --- |
| Control siRNA | 5'-UUAUGCCGAUCGCGUCACATT-3'  3'-TTAAUACGGCUAGCGCAGUGU-5' |
| FABP4 siRNA seq 1 | 5’-GACGUUGACCUGGACUGAAdTdT-3’  3’-UUCAGUCCAGGUCAACGUCdTdT-5’ |
| FABP4 siRNA seq 2 | 5’-GUGGGAUAUAUUGUUCAAAdTdT-3’  3’-UUUGAACAAUAUAUCCCACdTdT-5’ |

| Primers | Primer sequences |
| --- | --- |
| 18S | 5’CGCCGCTAGAGGTGAAATTC3’ (forward) and 5’TTGGCAAATGCTTTCGCTC3’ (reverse) |
| FABP4 | 5'-TGATGATCATGTTAGGTTTGGC-3' (forward) and 5'-TGGAAACTTGTCTCCAGTGAA-3' (reverse) |
| Mutated | caacaatatctttttgaacaatatatcccacaggcgacggtagagttcaatgcgaacttcagtccaggtcaa (forward)  Ttgacctggactgaagttcgcattgaactctaccgtcgcctgtgggatatattgttcaaaaagatattgttg (reverse) |

**Supplementary Table 8** siRNA and primer sequences used in the study
