# Supplementary material for: FABP4 as a key determinant of metastatic potential of ovarian cancer
Source: Nat Commun. 2018 Jul 26;9:2923. doi: 10.1038/s41467-018-04987-y (PMC6062524; doi:10.1038/s41467-018-04987-y)
Supplement: Supplementary file 2 — Description of Additional Supplementary Files [file 41467_2018_4987_MOESM2_ESM.pdf]

Description of Additional Supplementary Files

File Name: Supplementary Data 1
Description: RPPA log2 transformed data in HeyA8 MDR cells.
